# Histone Demethylase UTX Suppresses Tumor Cell Proliferation by Regulating Stress Granules

**DOI:** 10.1002/advs.202501990

**Published:** 2025-06-19

**Authors:** Xikai Liu, Xinran Liu, Mei Xue, Yushuo Xiao, Yuchen Chen, Rong Qiu, Di Wu, Yihao Zhou, Jiao Wang, Yan Yuan, Linwei Yu, Tianyi Shi, Yangkai Li, Hua Su, Hong Chen, Yong Liu, Kun Huang, Ling Zheng

**Affiliations:** ^1^ State Key Laboratory of Metabolism and Regulation in Complex Organisms Hubei Key Laboratory of Cell Homeostasis TaiKang Center for Life and Medical Sciences Frontier Science Center for Immunology and Metabolism College of Life Sciences Wuhan University Wuhan 430072 China; ^2^ School of Pharmacy Tongji Medical College and State Key Laboratory for Diagnosis and Treatment of Severe Zoonotic Infectious Diseases Huazhong University of Science and Technology Wuhan 430030 China; ^3^ Wuhan No.1 Hospital Tongji Medical College Huazhong University of Science and Technology Wuhan 430000 China; ^4^ Department of Thoracic Surgery Tongji Hospital Tongji Medical College Huazhong University of Science and Technology Wuhan 430030 China; ^5^ Department of Nephrology Union Hospital Tongji Medical College Huazhong University of Science and Technology Wuhan 430022 China

**Keywords:** endoplasmic reticulum stress, stress granule, tumorigenesis, TPR domain, UTX

## Abstract

UTX (also known as KDM6A) is a histone H3K27 demethylase that acts as an important tumor regulator. UTX has been reported to participate in genome‐wide histone modifications and gene expression in tumorigenesis and its mutations are identified in human cancers. Here, UTX is demonstrated to localize both in the cytoplasm and nucleus, notably, cytoplasmic UTX forms puncta and co‐localizes in stress granules (SGs) upon different stresses in vitro. Mechanistically, the TPR domain of UTX plays a critical role in regulating SG disassembly by interacting with G3BP1, the central hub of SG, to disrupt the scaffold network of SG under endoplasmic reticulum stress. Importantly, a clinical UTX mutation, D336G in TPR domain, increases cytoplasmic location of UTX, and stabilizes SG. While UTX^D336G^ promotes, WT UTX or UTX^TPR^ inhibits, cell growth and tumorigenesis by regulating SGs both in vitro and in nude mice, and such regulation is G3BP1 dependent. Together, the results suggest a novel cytoplasmic function of UTX as a negative regulator of SG homeostasis, which is involved in stress and disease states such as tumorigenesis.

## Graphical abstract

These findings indicate that cytoplasmic UTX forms puncta and co‐localizes in stress granules (SGs) upon various stresses. UTX TPR‐domain‐dependently and demethylase‐activity‐independently destabilize SGs by binding G3BP1, the SG hub protein, to disrupt SG network, thus affects tumorigenesis. D336G, a clinical UTX mutation in TPR domain, promotes tumorigenesis by stabilizing SGs in vitro and in nude mice.

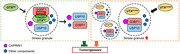

## 1 Introduction

Stress granules (SGs) are cytoplasmic membraneless organelles formed by proteins and mRNA through liquid‐liquid phase separation.^[^ ^1^ ^]^ Upon various external stimuli, such as oxidative stress, heat stress, or endoplasmic reticulum (ER) stress, SGs are quickly formed via phase separation, but can also be promptly disassembled after relieving stress.^[^ ^2^ ^]^ Structurally, SGs are non‐uniform with a highly packed core and a dynamic shell, while G3BP1 (GTPase‐activating protein SH3 domain‐binding protein 1) acts as the central node for SG networks.^[^ ^3^ ^]^ Although the formation of SGs is considered a transient response for cellular stresses, it plays important roles in many physiological processes, such as alternative splicing, protein synthesis, and cell growth.^[^ ^4^, ^5^ ^]^ Impaired assembly/disassembly of SGs are associated with multiple diseases, such as cancer,^[^ ^6^ ^]^ metabolic diseases,^[^ ^7^ ^]^ and neurodegenerative diseases.^[^ ^8^ ^]^

Histone demethylase UTX (ubiquitously transcribed tetratricopeptide repeat on chromosome X, also known as KDM6A), mainly functioned in the nucleus by removing di‐ and tri‐methyl groups on H3K27,^[^ ^9^ ^]^ plays roles in various biological processes, such as controlling cell fate,^[^ ^10^ ^]^ muscle regeneration,^[^ ^11^ ^]^ embryonic, cardiac, and cerebral development.^[^ ^12^, ^13^, ^14^ ^]^ Moreover, UTX is also involved in multiple diseases, including acute liver injury,^[^ ^15^ ^]^ diabetic kidney disease,^[^ ^16^, ^17^, ^18^ ^]^ and cancers.^[^ ^19^, ^20^, ^21^ ^]^ UTX consists of an N‐terminal TPR (tetratricopeptide repeat) domain containing 8 TPRs (www.uniprot.org), an intrinsically disordered region (IDR), and a C‐terminal JmjC (Jumonji C) domain for its demethylase activity.^[^ ^22^ ^]^ Recently, UTX has been reported to mediate genome‐wide histone modifications and gene expression by recruiting histone methyltransferase MLL4, through phase separation by its IDR region in the nucleus.^[^ ^19^ ^]^ However, some studies observe the existence of UTX in the cytoplasm.^[^ ^22^, ^23^, ^24^ ^]^ Furthermore, cancer‐derived UTX TPR mutations, G137V (UTX^G137V^) and D336G (UTX^D336G^), impair its interaction with MLL3/4, leading to increased cytoplasmic location of UTX.^[^ ^25^ ^]^ However, the functions of cytoplasmic UTX remain unclear.

Here, we report a novel SG‐dynamics‐regulating role of UTX. Upon ER stress, cytoplasmic UTX forms puncta co‐localized with SGs, which TPR‐domain‐dependently and demethylase‐activity‐independently destabilize SGs. Mechanistically, cytoplasmic UTX is recruited to SGs, where it interacts with the nuclear transport factor 2‐like (NTF2L) domain of the SG hub protein G3BP1 to disrupt the SG network. A clinical UTX mutation that increased its cytoplasmic location,^[^ ^25^ ^]^ UTX^D336G^, stabilizes SGs in vitro and in nude mice. While UTX^D336G^ promotes, UTX or UTX^TPR^ inhibits cell growth and tumorigenesis by regulating SGs, and such regulation is G3BP1 dependent. Together, our results reveal a TPR‐domain‐dependent and demethylase‐activity‐independent tumorigenesis‐suppressing function of UTX through regulating the homeostasis of stress granules.

## 2 Results

### 2.1 Cytoplasmic UTX Forms Puncta that Localize with Stress Granules Upon Stresses

**Figure 1:**
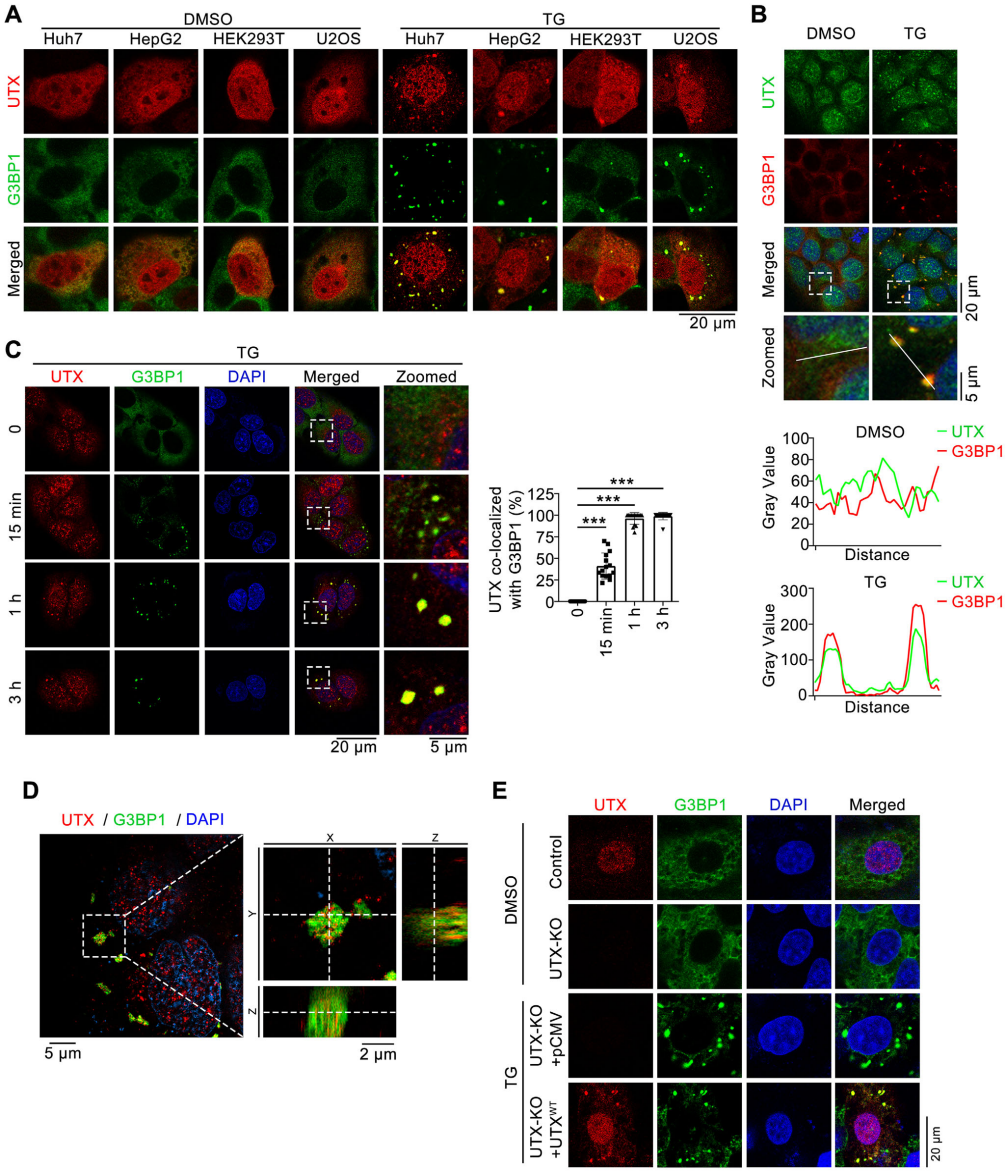
Cytoplasmic UTX forms puncta and localizes in stress granules of thapsigargin‐treated cells. A) Representative images of UTX (red) and G3BP1 (green) in UTX overexpressed Huh7, HepG2, HEK293T, or U2OS cells treated with vehicle (DMSO) or thapsigargin (TG). Huh7 cells, 1 µm TG, 1 h; HepG2 cells, 5 µm TG, 4 h; HEK293T cells, 2.5 µm TG, 1 h; U2OS cells, 5 µm TG, 1 h; Scale bar, 20 µm. B) Representative images of endogenous UTX (green; antibody from Abcam) and G3BP1 (red) (top panel), with plot analysis for the green and red pixel intensities along the indicated arrows in the zoomed pictures (bottom panel). Huh7 cells, 1 µm TG, 1 h. Scale bar, 20 µm. Magnification of the demarcated region was shown as the inset from the merged images, with the inset scale bar representing 5 µm. C) Representative images of UTX (red; antibody from Abcam) and G3BP1 (green) (left panel) with quantification result of the percentage of cytoplasmic UTX puncta co‐localized with G3BP1^+^ stress granules (right panel) in Huh7 cells treated with 1 µm TG for indicated time. Scale bar, 20 µm. Shown also are magnifications of the indicated inset regions, with the inset scale bar representing 5 µm. Data are shown as the mean ± SD; *n* = 15 image fields per group from three different samples; ***p < 0.001 (analyzed by one‐way ANOVA). D) Z‐stack projection of the representative image of co‐localized endogenous UTX (red) and G3BP1 (green) in Huh7 cells under 1 µm TG treatment for 1 h (left panel; scale bar, 5 µm); magnified orthogonal sectioning view of regions in the insert box (right panel; scale bar, 2 µm). DAPI, blue, stained nuclei. E) Representative images of UTX (red) and G3BP1 (green) in stable knockout UTX HepG2 cells transfected with empty vector (pCMV) or UTX WT plasmid (UTX^WT^) treated with 5 µm TG for 4 h. Scale bar, 20 µm. All results are representative for at least three independent experiments, with similar results obtained.

UTX is a well‐known protein with histone H3K27me2/me3 demethylase activity mostly located in the nucleus.^[^ ^9^ ^]^ However, in UTX overexpressing Huh7 cells, fair amount of UTX was found in the cytoplasm (**Figure** **1**A; Figure S1, Supporting Information); consistently, its cytoplasmic location was also observed in UTX overexpressing HepG2, HEK293T and U2OS cells under normal conditions (Figure 1A; Figure S1, Supporting Information). Interestingly, cytoplasmic UTX also formed puncta in these UTX overexpressing cells upon thapsigargin (TG) treatment (Figure 1A). Because TG has been reported to induce stress granules (SGs) formation in cultured cells,^[^ ^5^ ^]^ we examined whether these UTX puncta are co‐localized with SGs. Indeed, cytoplasmic UTX puncta co‐localized with G3BP1 puncta, the major hub protein and marker for SGs, in UTX overexpressing cells under TG treatment (Figure 1A). Importantly, endogenous UTX also formed cytoplasmic puncta and co‐localized with G3BP1‐positive SGs at 1 h after TG treatment in Huh7 cells (Figure 1B). Furthermore, a time course study demonstrated that while only 41% endogenous cytoplasmic UTX puncta were co‐localized with SGs at 15 min after TG treatment, about 96% cytoplasmic UTX puncta were co‐localized with SGs at 1 h and lasted for more than 3 h upon TG treatment (Figure 1C). To observe details of the distribution pattern of UTX and G3BP1 within SGs, we applied the super‐resolution structured illumination microscopy (SIM) and observed that UTX was non‐uniformly intercalated with G3BP1 (Figure 1D). Consistent findings were also obtained in a parallel study using another commercial UTX antibody (Figure S2, Supporting Information). Furthermore, we overexpressed UTX in UTX knockout HepG2 cells, and also found formation of cytoplasmic UTX puncta co‐localizing with G3BP1 positive SGs under TG treatment (Figure 1E). Besides TG, another ER stress inducer DTT,^[^ ^26^ ^]^ as well as additional well‐known SG inducers such as sodium arsenite (SA) and heat shock (HS), were also applied.^[^ ^27^ ^]^ As expected, endogenous cytoplasmic UTX also formed puncta and co‐localized with G3BP1 positive SGs under DTT‐ or SA‐ or HS‐treatment (Figure S3, Supporting Information).

Next, we investigated whether the appearance of UTX cytoplasmic puncta depends on SGs formation. Because in some cell lines, SG formation requires the core proteins G3BP1 and G3BP2,^[^ ^3^ ^]^ a G3BP1 and G3BP2 double knockout (dKO) U2OS cell line was constructed (Figure S4A, Supporting Information). In TG‐treated dKO U2OS cells, SGs failed to form as demonstrated by staining for G3BP1 and another SG marker DDX3X (DEAD box protein 3, X‐chromosomal);^[^ ^28^ ^]^ meanwhile the cytoplasmic UTX puncta were not observed (Figure S4B,C, Supporting Information).

**Figure 2:**
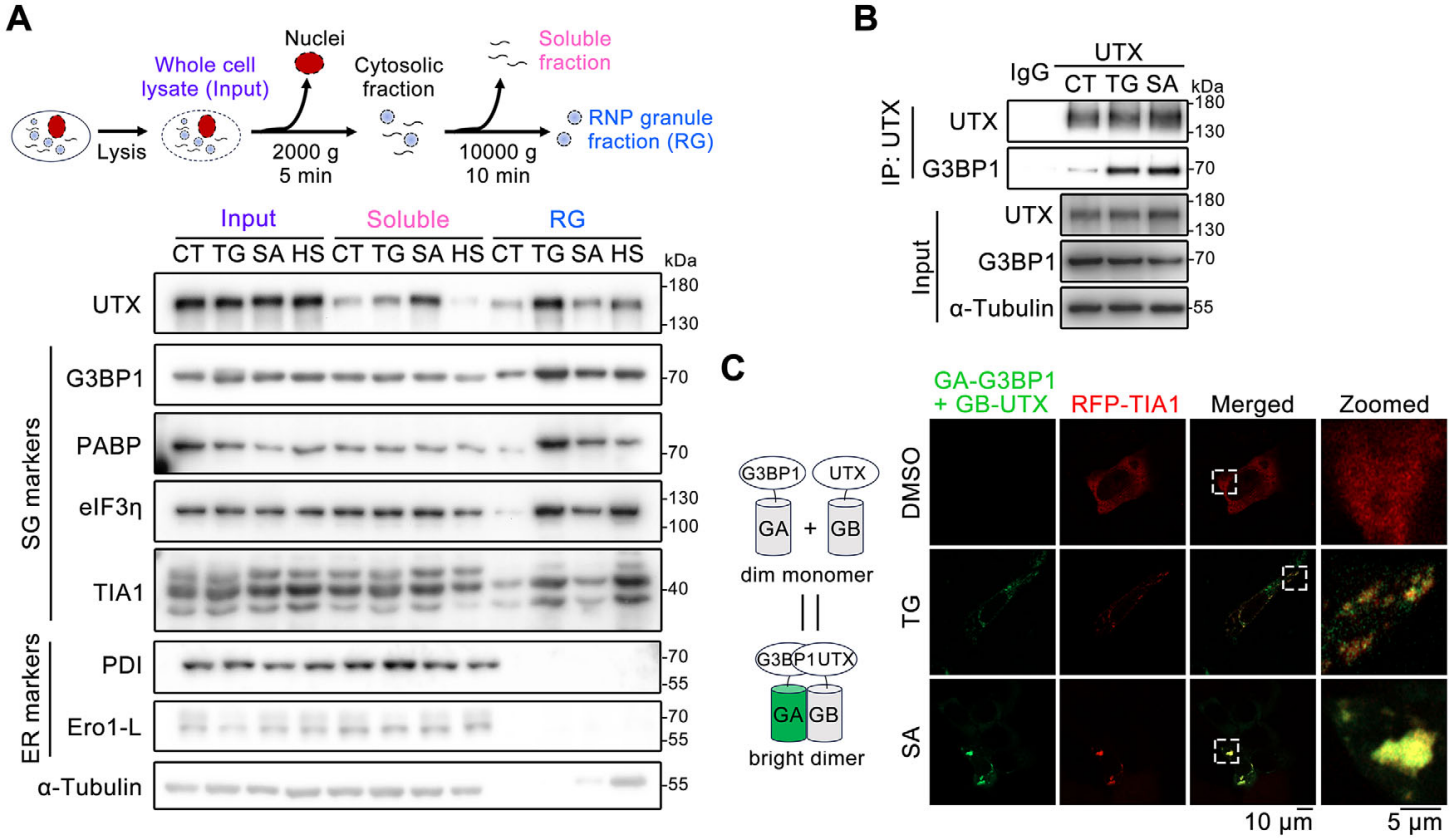
Enhanced association of cytoplasmic UTX with stress granule components upon stresses. A) A diagram for the subcellular fractionation process is shown in the top panel. Immunoblot analysis of indicated proteins in the whole cell lysates (input), nucleus‐free soluble fraction (soluble) and insoluble ribonucleoprotein granule fraction (RG) of Huh7 cells (bottom panel); CT, control; TG, 1 µm for 1 h; SA (sodium arsenite), 500 µm for 1 h; HS (heat shock), 43 °C for 1 h. B) Cytoplasmic fraction of Huh7 cells was subjected to immunoprecipitation with UTX antibody. Immunoblot analysis of the indicated proteins with α‐tubulin or IgG as the loading control. C) Diagram for the design of dimerization‐dependent fluorescent protein (ddFP) system between UTX and G3BP1 (left panel) with representative images of live HepG2 cells transiently transfected with GA‐G3BP1, GB‐UTX, and RFP‐TIA1, with or without TG (5 µm, 4 h)‐ or SA (500 µm, 1 h)‐treatment. Scale bar, 10 µm. Shown also are magnifications of the indicated inset regions, with the inset scale bar representing 5 µm. Results are representative for at least three independent experiments, with similar results obtained.

To seek biochemical evidence that cytoplasmic UTX is localized within SGs under different stresses, we fractionated the enriched cytoplasm into soluble fraction and SG‐containing insoluble ribonucleoprotein granule (RG) fraction.^[^ ^29^, ^30^ ^]^ Well‐recognized SG markers, such as G3BP1, PABP1 (polyadenylate‐binding protein 1), eIF3η (eukaryotic translation initiation factor 3 subunit B) and TIA1 (T‐cell‐restricted intracellular antigen‐1) were significantly enriched in the RG fraction but not in the soluble fraction in Huh7 cells upon TG‐, SA‐ or HS‐stress; while ER lumenal proteins, such as PDI (protein disulfide‐isomerase) and Ero1‐L (endoplasmic reticulum oxidoreductin‐1‐like protein) were enriched only in the soluble fraction with or without stress (**Figure** **2**A), indicating successful fractionation. As expected, significantly enriched UTX was found in the RG fraction upon different stresses at the SG formation stage (Figure 2A). In addition, co‐immunoprecipitation (co‐IP) detected significantly enhanced binding of G3BP1 with endogenous UTX in the RG fraction from TG‐ or SA‐treated Huh7 cells (Figure 2B). By using the dimerization‐dependent fluorescent protein (ddFP) reporter system,^[^ ^31^ ^]^ we investigated whether there are inter‐molecular contacts between UTX and G3BP1 in live cells by co‐expressing GA‐G3BP1 and GB‐UTX. No green fluorescence was detected in untreated cells, while bright green fluorescence was observed in TG‐ or SA‐treated HepG2 cells (Figure 2C), suggesting a close contact between G3BP1 and UTX in cytoplasm upon stresses; moreover, these bright green fluorescence signals were partially co‐localized with another well‐known SG marker TIA1 (RFP‐labeled) (Figure 2C), suggesting a UTX‐G3BP1 interaction within SGs. Consistently, binding between endogenous G3BP1 and UTX was also found, and was further enhanced upon TG treatment in primary mouse hepatocytes (normal cells) (Figure S5, Supporting Information).

### 2.2 The TPR Domain of UTX is Involved in SG Disassembly

**Figure 3:**
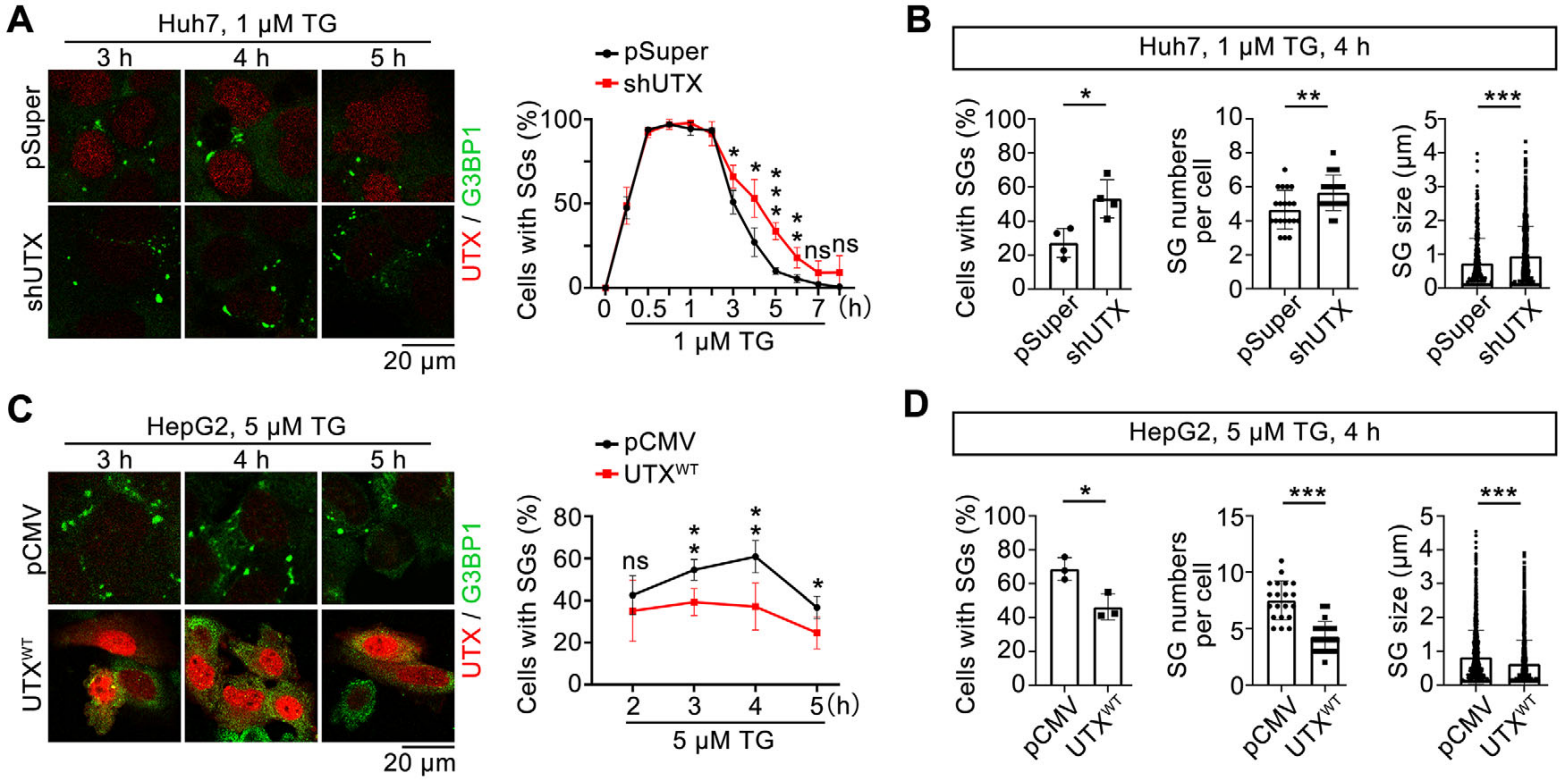
UTX accelerates thapsigargin‐induced stress granule disassembly. A) Representative images of UTX (red) and G3BP1 (green) (left) with quantification of the percentage of cells with SG (right) in Huh7 cells transfected with pSuper (control) or shUTX plasmid under 1 µm TG treatment for indicated time. Scale bar, 20 µm. B) Quantitative results of the percentage of cells with SG (left, *n* = 4 independent samples per group), number of SG per cell (middle, *n* = 20 cells per group collected from four independent samples), and SG size (right, 16 images per group collected from four independent samples) in Huh7 cells transfected with pSuper or shUTX plasmid under 1 µm TG for 4 h. C) Representative images of UTX (red) and G3BP1 (green) (left) with quantification of the percentage of cells with SG (right) in HepG2 cells transfected with empty vector (pCMV) or UTX wildtype (UTX^WT^) plasmid under 5 µm TG treatment for indicated time. Scale bar, 20 µm. D) Quantitative results of the percentage of cells with SG (left, *n* = 3 independent samples per group), number of SG per cell (middle, *n* = 20 cells per group collected from three independent samples), and SG size (right, 12 images per group collected from three independent samples) in HepG2 cells transfected with empty vector (pCMV) or UTX wildtype (UTX^WT^) plasmid under 5 µm TG for 4 h. All results are representative for at least three independent experiments, with similar results obtained. Data are shown as the mean ± SD, and analyzed by two‐tailed Student's *t*‐test. *p < 0.05, **p < 0.01, ***p < 0.001; ns, not significant.

As a transient dynamic membraneless organelle, the existence of SGs involves both assembly and disassembly processes.^[^ ^27^ ^]^ We found that the dynamics of SGs in TG‐treated Huh7 cells (Figure S6A,B, Supporting Information) or HepG2 cells (Figure S6D,E, Supporting Information) were in a dosage‐ or time‐dependent manner, which were fast assembly and disassembly, consistent with previously reports.^[^ ^5^, ^32^ ^]^ Moreover, to investigate whether SG disassembly is increased by reliving the stress, we washed out TG with fresh media at the point of maximum SG formation, and found no difference at the early SG disassembly stage (Figure S6C,F, Supporting Information). Therefore, the 1 or 5 µm TG treatment without washing out was chosen to study whether UTX regulates the dynamics of SGs in Huh7 and HepG2 cells, respectively. Transiently knockdown UTX by shRNAs prolonged SG disassembly in Huh7 cells (**Figure** **3**A,B); consistently, transiently overexpressing UTX accelerated the disassembly of SG in HepG2 cells (Figure 3C,D). However, in either cell lines, the transcription and protein levels of G3BP1 were unaffected, with or without TG treatment (Figure S7, Supporting Information). Besides, transiently overexpressing UTX also accelerated the disassembly of SG in DTT‐ or SA‐ or HS‐treated HepG2 cells (Figure S8, Supporting Information).

**Figure 4:**
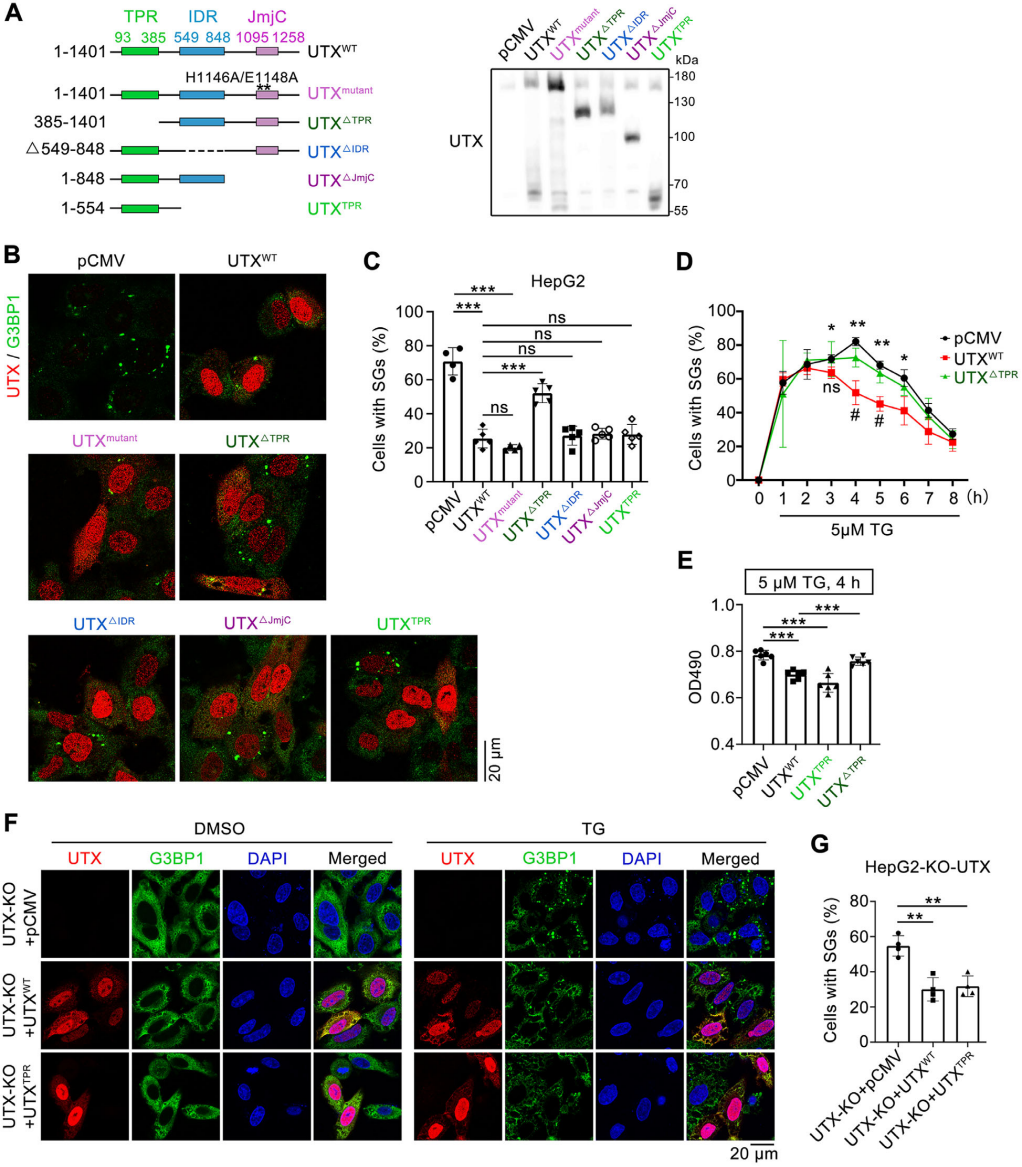
UTX promotes thapsigargin‐induced stress granule disassembly through its TPR domain. A) The plasmid constructs of wildtype, enzymatic mutation, and different functional domain deletion of UTX (left panel) used in the present study, and verified by Western blots (right panel). B,C) Representative images of UTX (red) and G3BP1 (green) (B) with quantitative results of the percentage of cells with SGs (C) in HepG2 cells transfected with indicated plasmid under 5 µm TG treatment for 4 h. Scale bar, 20 µm. *n* = 4–5 image fields per group. D) Quantification of the percentage of cells with SG in HepG2 cells transfected with empty vector (pCMV) or wildtype UTX (UTX^WT^) or TPR‐domain deleted UTX (UTX^△TPR^) plasmid under 5 µm TG treatment for indicated time. * comparison with pCMV and UTX^WT^; # comparison with UTX^WT^ and UTX^△TPR^. *n* = 3 per group. E) MTT assays in HepG2 cells transfected with empty vector (pCMV), UTX^WT^, UTX^TPR^, or UTX^△TPR^ under 5 µm TG treatment for 4 h. *n* = 6 per group. F,G) Representative images of UTX (red) and G3BP1 (green) (F) with quantitative results (G) of the percentage of cells with SGs in UTX knockout HepG2 cells transfected with empty vector (pCMV), UTX^WT^ or UTX^TPR^, and treated with vehicle (DMSO) or 5 µm TG for 4 h. Scale bar, 20 µm. *n* = 4 per group. All results are representative for at least three independent experiments, with similar results obtained. Data are shown as the mean ± SD, and analyzed by one‐way ANOVA. *p < 0.05, **p < 0.01, ***p < 0.001; ns, not significant.

UTX has multiple functional domains,^[^ ^33^ ^]^ we next investigated which domain may play an important role in regulating SGs disassembly. Double point mutation (H1146A/E1148A, UTX^mut^) that abolish its H3K27 demethylase activity,^[^ ^34^ ^]^ as well as UTX domain truncated mutants including UTX^△TPR^, UTX^△IDR^, UTX^△JmjC^, or TPR domain only (UTX^TPR^), were used (**Figure** **4**A). At 4 h after TG treatment, it was the TPR domain, but not the IDR domain nor the JmjC domain or UTX^mut^, that was necessary and sufficient to promote SGs disassembly in HepG2 cells (Figure 4B,C); consistently, time course study demonstrated that while UTX accelerated the disassembly of SGs, UTX^△TPR^ lost this ability (Figure 4D). Since UTX regulates tumorigenesis,^[^ ^19^ ^]^ we examined whether the cell growth rate is affected by UTX or its TPR domain. MTT assays suggested that overexpression of either UTX^WT^ or UTX^TPR^, but not UTX^△TPR^, inhibited cell proliferation in HepG2 cells under the TG treatment (Figure 4E). Besides, overexpressing of UTX^WT^ or UTX^TPR^ in UTX knockout HepG2 cells also promoted SGs disassembly under TG stress (Figure 4F,G). To investigate whether UTX^TPR^ affects its enzyme activity, H3K27me2/me3 levels, as well as H3K27ac, H3K4me3, and DNA methylation levels which have been shown affected by UTX,^[^ ^35^ ^]^ were evaluated. As expected, UTX^WT^ significantly down‐regulated, while UTX^mut^ showed no effect on H3K27me2/me3 levels; meanwhile, UTX^TPR^ also showed no effect on H3K27me2/me3 levels in HepG2 and HEK293T cells (Figure S9A,C, Supporting Information). Furthermore, none of UTX^WT^, UTX^mut^, or UTX^TPR^ affected the H3K27ac, H3K4me3, and 5mC levels in HepG2 and/or HEK293T cells (Figure S9B–D, Supporting Information). Collectively, these results indicated that UTX, TPR‐domain‐dependently, enzyme‐activity‐independently, promotes SGs disassembly and possibly affects cell growth.

### 2.3 UTX TPR Domain Interacts with G3BP1 NTF2L Domain to Block SG Network and Cause SG Disassembly

**Figure 5:**
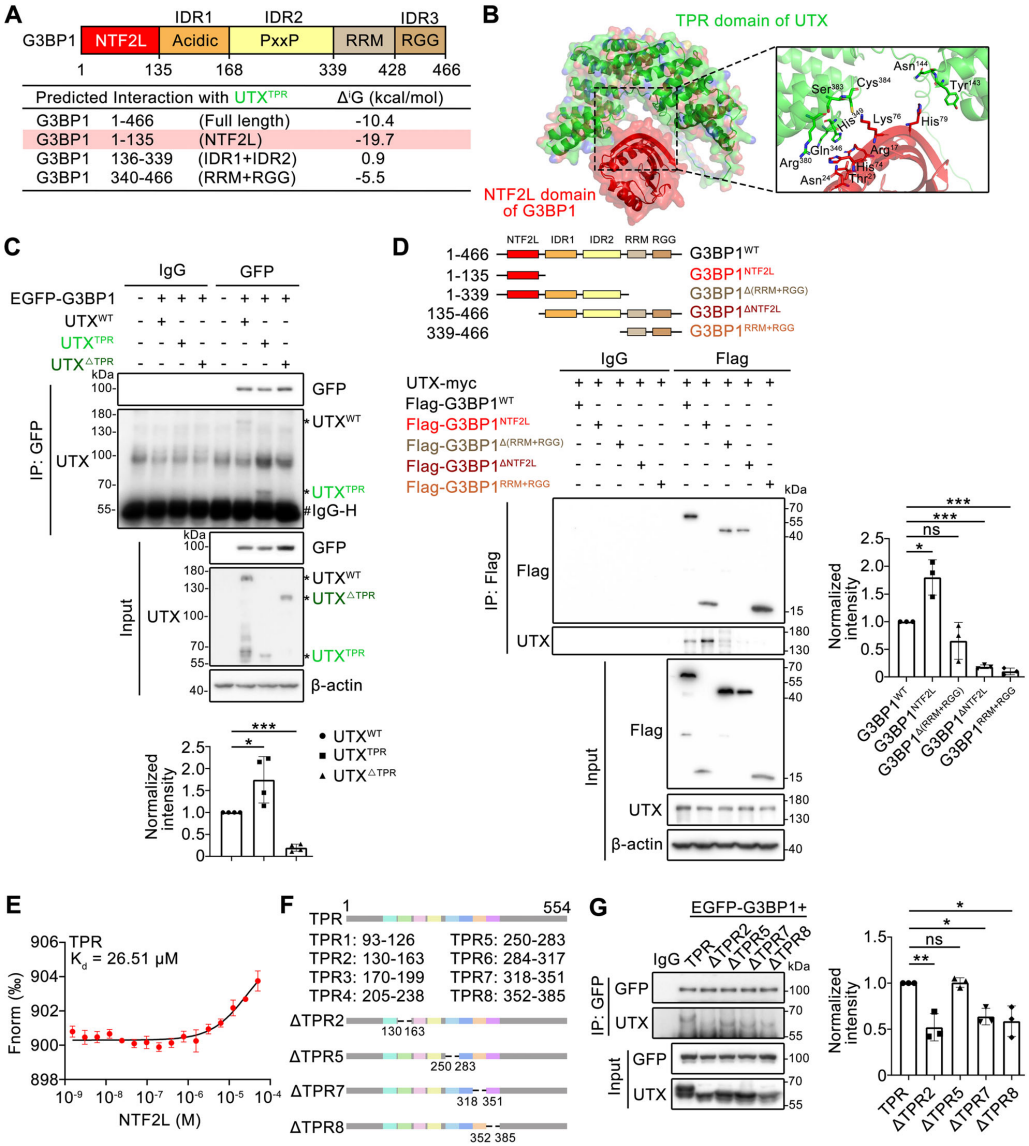
UTX interacts with the NTF2L domain of G3BP1. A) Schematic representation of different G3BP1 domains (up panel) and potential interaction with UTX^TPR^ predicted by AlphaFold (bottom panel). B) Interaction between the TPR domain of UTX and NTF2L domain of G3BP1 predicted by AlphaFold and the interaction interface residues are marked on the right. C) Co‐immunoprecipitation assays for G3BP1 and UTX in HEK293T cells transfected with EGFP‐tagged G3BP1 and different domain truncated UTX. * indicates corresponding bands of WT or truncated domains of UTX; # indicates IgG heavy chain. *n* = 4 per group. D) Co‐immunoprecipitation assays for UTX and G3BP1 in HEK293T cells transfected with Myc‐tagged UTX and different truncated domains of G3BP1. *n* = 3 per group. E) MST analysis of the interaction between the TPR and NTF2L domains. Recombinant TPR (50 nm) was incubated with increasing concentrations of NTF2L proteins. F) Schematic representation of different TPR domains. G) Co‐immunoprecipitation assays for G3BP1 and TPR domains in HEK293T cells transfected with EGFP‐tagged G3BP1 and different domain‐deleted of TPR domain. *n* = 3 per group. All results are representative for at least three independent experiments, with similar results obtained. Data are shown as the mean ± SD, and analyzed by one‐way ANOVA. *p < 0.05, **p < 0.01, ***p < 0.001; ns, not significant.

G3BP1 has five domains, nuclear transport factor 2 like (NTF2L) domain, acidic domain (IDR domain 1), PxxP domain (IDR domain 2), RNA recognition motif, and RGG domain (or IDR domain 3) (**Figure** **5**A).^[^ ^36^ ^]^ Since UTX binds to G3BP1 as shown in Figure 2B, we further mapped the interaction domains between these two proteins. AlphaFold was applied and predicted strong interactions between the TPR domain of UTX and the NTF2L domain of G3BP1, as well as the key interacting residues between these two domains (Figure 5A,B). Further domain mapping assays revealed that UTX TPR‐domain‐dependently interacted with G3BP1, mainly with its NTF2L domain (Figure 5C,D). Microscale thermophoresis (MST) assay, using purified UTX TPR domain and G3BP1 NTF2L domain, further demonstrated binding between these two domains (Figure 5E). Because AlphaFold predicted key interacting residues for UTX TPR domain and G3BP1 NTF2L domain reside on TPR2, 7 and 8, their truncations as well as TPR5 truncation were constructed (Figure 5F). Co‐IP experiments confirmed that TPR2, 7 and 8, but not TPR5, were the key TPRs that bind G3BP1 (Figure 5G).

**Figure 6:**
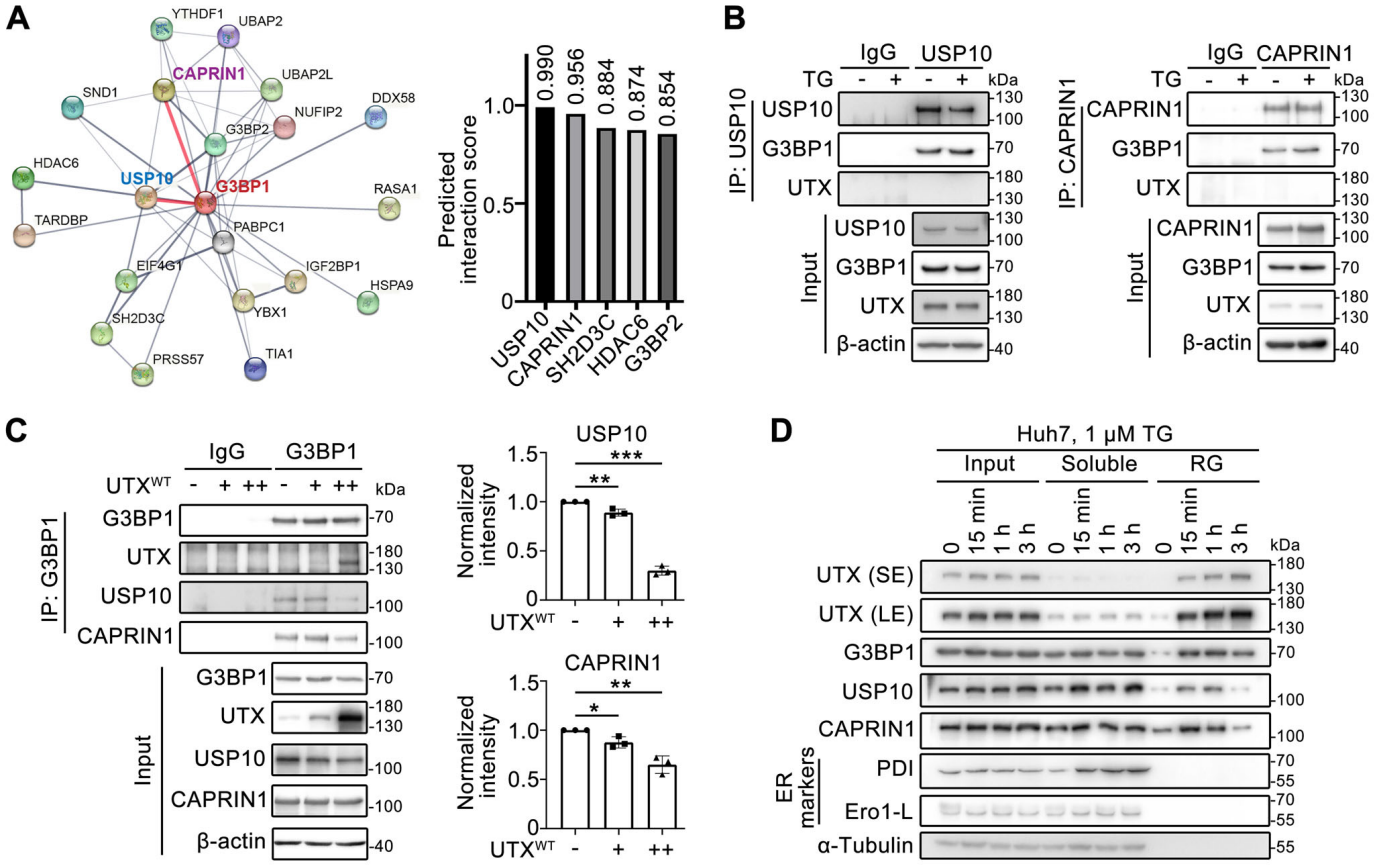
UTX disassembles stress granules by blocking the G3BP1 interaction network. A) STRING network from GeneCards (https://www.genecards.org/) provides G3BP1 interaction protein network (left panel) with the five top‐scored proteins shown in the right panel. B) Co‐immunoprecipitation assays for USP10 (left) or CAPRIN (right) with G3BP1 or UTX in Huh7 cells without or with 1 µm TG for 1 h. C) Co‐immunoprecipitation assays for G3BP1 with USP10, CAPRIN, or UTX in HEK293T cells transfected with increasing amounts of UTX^WT^ plasmid (left panel) and quantification results (right panel). *n* = 3 per group. Results are shown as mean ± SD., and analyzed by one‐way ANOVA. *p < 0.05, **p < 0.01, ***p < 0.001. D) Immunoblot analysis of the indicated proteins from whole cell lysates (input), soluble fraction without nucleus (soluble) and the insoluble ribonucleoprotein granule (RG) fraction in Huh7 cells treated with 1 µm TG for indicated time. All results are representative for at least three independent experiments, with similar results obtained.

As the master hub protein for SGs assembly, the homeostasis of SGs is maintained by the interaction networks centered by G3BP1 with other SGs proteins,^[^ ^3^ ^]^ and the NTF2L domain of G3BP1 is the scaffold for these interactions.^[^ ^37^ ^]^ Thus, we speculated that UTX may compete for NTF2L binding to affect SGs disassembly. In the complex interaction network of G3BP1, USP10 and CAPRIN1 are two top binding partners as the STRING suggested (**Figure** **6**A), which interact with the NTF2L domain and regulate SGs stability.^[^ ^37^, ^38^, ^39^ ^]^ Co‐IP assays suggested that USP10 and CAPRIN1 strongly interacted with G3BP1 but not with UTX, with or without TG treatment (Figure 6B). Furthermore, interaction between G3BP1 and USP10 or CAPRIN1 was dose‐dependently disrupted by UTX overexpression in HEK293T cells (Figure 6C). Consistently, a time course study on TG‐treated Huh7 cells using fractionated soluble and RG parts demonstrated a gradual UTX enrichment in the RG part; while the levels of USP10 and CAPRIN1 were initially enriched in the RG part within 15 min after treatment (assembly phase), but dramatically reduced at 3 h after treatment (disassembly phase) (Figure 6D). Moreover, G3BP1 was enriched in the RG part within 15 min after treatment and remained stable at 3 h after treatment (Figure 6D). Collectively, these results suggested that UTX is recruited to SGs upon stress and competes with G3BP1‐binding proteins to disrupt the SG scaffold networks and dissemble SGs.

### 2.4 Clinical Mutations of UTX Promote SG Formation

**Figure 7:**
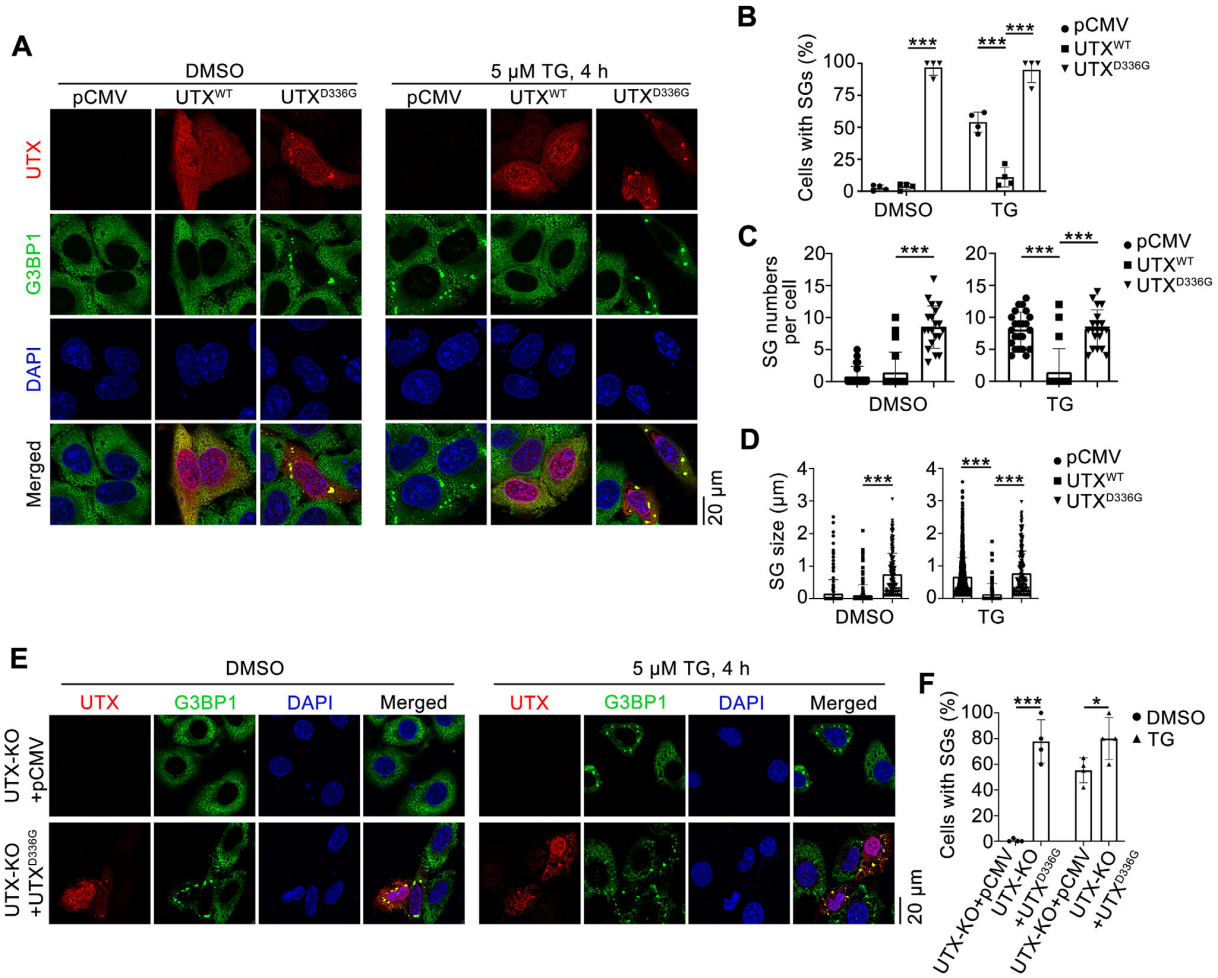
Clinical UTX mutation stabilizes SG under thapsigargin treatment. A–D) Representative images of UTX (red) and G3BP1 (green) (A) with quantitative results of the percentage of cells with SGs (B, *n* = 4 per group), number of SG per cell (C, *n* = 20 cells per group collected from four independent samples), and size of SG (D, 12 images per group collected from four independent samples) in HepG2 cells transfected with empty vector (pCMV), UTX^WT^ or UTX^D336G^, under vehicle (DMSO) or 5 µm TG treatment for 4 h. Scale bar, 20 µm. E,F) Representative images of UTX (red) and G3BP1 (green) (E) with quantitative results of the percentage of cells with SG (F) in UTX stable knockout HepG2 cells transfected with empty vector (pCMV) or UTX^D336G^ under vehicle (DMSO) or 5 µm TG treatment for 4 h. Scale bar, 20 µm. All results are representative for at least three independent experiments, with similar results obtained. Data are shown as the mean ± SD, and analyzed by one‐way ANOVA. *p < 0.05, ***p < 0.001.

Two clinical UTX mutations, G137V and D336G in TPR domain, are reported to be preferentially cytoplasmic localized because they impair the interaction of UTX with the MLL3/4 complexes.^[^ ^25^ ^]^ To gain insights on the effects of these clinical mutations on SG formation, they were transiently overexpressed in HepG2 cells. Since the UTX^G137V^ is reported unstable^[^ ^25^ ^]^ and confirmed by our own observations (data not shown), UTX^D336G^ was chosen for the following studies. The percentage of cells with SGs, as well as the number and the size of SGs, were significantly increased in UTX^D336G^ overexpressed cells, with or without TG treatment (**Figure** **7**A–D). Besides, overexpression of UTX^D336G^ in UTX knockout HepG2 cells also showed similar effects (Figure 7E,F). Next, we examined whether the observed cytoplasmic puncta in UTX^D336G^ overexpressed cells are SGs, G3BP1/G3BP2 double knockout (dKO) U2OS cells were used, and UTX^D336G^ mutant failed to form cytoplasmic puncta in the dKO cells (Figure S10, Supporting Information).

### 2.5 UTX^D336G^ Promotes Tumorigenesis by Stabilizing SG in Nude Mice

**Figure 8:**
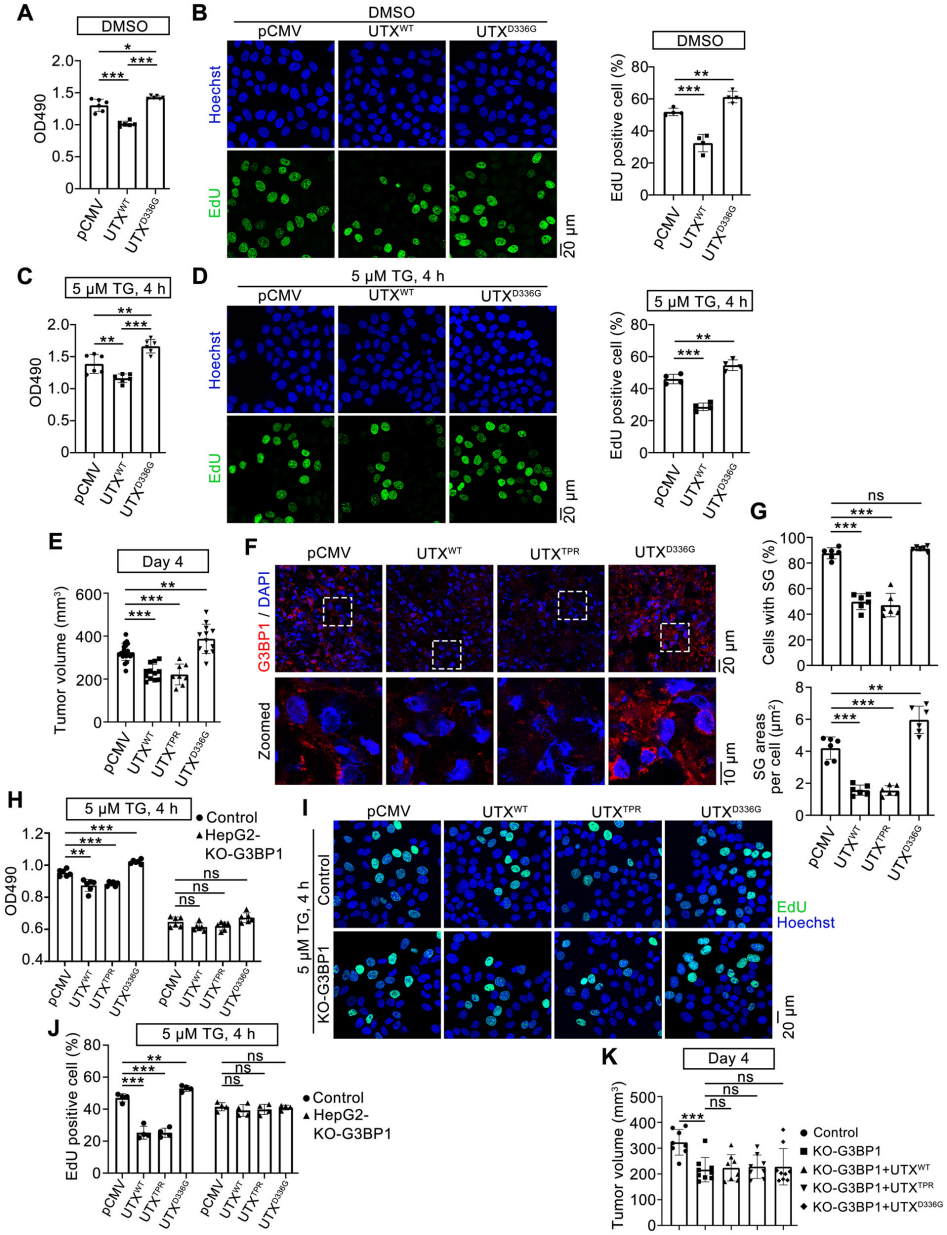
Clinical UTX mutation promotes tumorigenesis by stabilizing SG. A,C) MTT assays in HepG2 cells transfected with empty vector (pCMV), UTX^WT^, or UTX^D336G^ under vehicle (DMSO; A) or 5 µm TG treatment for 4 h (C). *n* = 6 per group. B,D) Representative images (left) with quantitative results (right) of EdU assay in HepG2 cells transfected with empty vector (pCMV), UTX^WT^, or UTX^D336G^ under vehicle (DMSO; B) or 5 µm TG treatment for 4 h (D). EdU positive cells (green); Hoechst stained nuclei (blue). Scale bar, 20 µm. *n* = 4 per group. E) Volume of xenograft tumors at day 4 from indicated groups. pCMV, *n* = 20; UTX^WT^, *n* = 12; UTX^TPR^, *n* = 8; UTX^D336G^, *n* = 12. F,G) Representative images of G3BP1 staining (red, F) with quantitative results of the percentage of cells with SGs (G, up) and SG areas per cell (G, bottom) in xenograft tumors. G3BP1^+^, red; DAPI, blue, stained nuclei. *n* = 6 mice per group. Scale bar, 20 µm. Shown also are magnifications of indicated inset regions, with the inset scale bar representing 10 µm. H) MTT assays in G3BP1 knockout HepG2 cells transfected with empty vector (pCMV), UTX^WT^, UTX^TPR^, and UTX^D336G^ under 5 µm TG treatment for 4 h. *n* = 6 per group. I,J) Representative images (I) with quantitative results (J) of EdU assay in the control or G3BP1 knockout HepG2 cells transfected with empty vector (pCMV), UTX^WT^, UTX^TPR^, and UTX^D336G^ under 5 µm TG treatment for 4 h. Scale bar, 20 µm. *n* = 4 per group. K) Volume of xenograft tumors at day 4 from indicated groups injected with control or G3BP1 knockout HepG2 cells which are transfected with empty vector (pCMV), UTX^WT^, UTX^TPR^, and UTX^D336G^. Control, *n* = 8; KO‐G3BP1, *n* = 9; KO‐G3BP1 + UTX^WT^, *n* = 8; KO‐G3BP1 + UTX^TPR^, *n* = 8; KO‐G3BP1 + UTX^D336G^, *n* = 9. All cell culture results are representative for at least three independent experiments, with similar results obtained. Data are shown as the mean ± SD, and analyzed by one‐way ANOVA. *p < 0.05, **p < 0.01, ***p < 0.001; ns, not significant.

To investigate whether the effects of UTX^WT^, UTX^TPR^, and UTX^D336G^ on SGs also contribute to tumorigenesis, in vitro MTT and EdU assays in TG‐treated HepG2 cells and in vivo xenografted tumor growth in nude mice were used. MTT and/or EdU assays suggested that while overexpression of UTX^WT^ inhibited, overexpression of UTX^D336G^ significantly promoted cell proliferation in HepG2 cells, with or without TG treatment (**Figure** **8**A–D; Figure S11A, Supporting Information). Moreover, similar effects of UTX^WT^, UTX^△TPR^, UTX^TPR^, and UTX^D336G^ on cell proliferation were observed in UTX knockout HepG2 cells under TG treatment (Figure S11B, Supporting Information).

Next, we investigated the effects of UTX^WT^, UTX^TPR^, and UTX^D336G^ on xenografted tumor growth in nude mice at 4 days after cell injection. Compared with the control xenografts, the tumor volumes were significantly increased in UTX^D336G^ xenografts, while were significantly decreased in UTX^WT^ or UTX^TPR^ xenografts (Figure 8E). Further immunofluorescence staining showed that compared with those of the control xenografts, the percentage and areas of SGs were significantly decreased in UTX^WT^ and UTX^TPR^ xenografts, while the areas of SGs were significantly increased in UTX^D336G^ xenografts (Figure 8F,G). Similar results were demonstrated at 10 days after cell injection (Figure S12, Supporting Information). Since HepG2 cells could not survive under G3BP1/G3BP2 double knockout (data not shown), G3BP1 knockout HepG2 cells, in which SG was greatly diminished, were constructed (Figure S13A,B, Supporting Information). Thus, MTT and EdU assays were performed on G3BP1 knockout HepG2 cells to investigate whether the cell growth‐regulating role of UTX depends on SG formation. G3BP1 knockout significantly reduced cell growth under TG treatment, indicating SGs do help cell survival (Figure 8H–J). Furthermore, compared with those in control cells, in G3BP1 knockout HepG2 cells, similarly overexpression of UTX^WT^, UTX^TPR^ or UTX^D336G^ does show attenuated effects on cell proliferation (Figure 8H–J). Besides, xenografted tumor growth in nude mice were also performed using HepG2 cells that overexpress UTX^WT^, UTX^TPR^, or UTX^D336G^ in G3BP1 knockout background at 4 days after cell injection. Compared with the control xenografts, G3BP1 was completely knockout in G3BP1‐KO xenografts as suggested by immunofluorescence staining (Figure S13C, Supporting Information). Consistent with in vitro proliferation results, tumor volumes were significantly decreased in G3BP1‐KO xenografts compared with the control xenografts (Figure 8K). Furthermore, compared with the control G3BP1‐KO xenografts, neither UTX^WT^/UTX^TPR^ nor UTX^D336G^ xenografts in G3BP1‐KO background showed significant changes in tumor volumes (Figure 8K). Collectively, these results indicated that UTX regulates tumor growth, at least partially, through regulating SGs.

**Figure 9:**
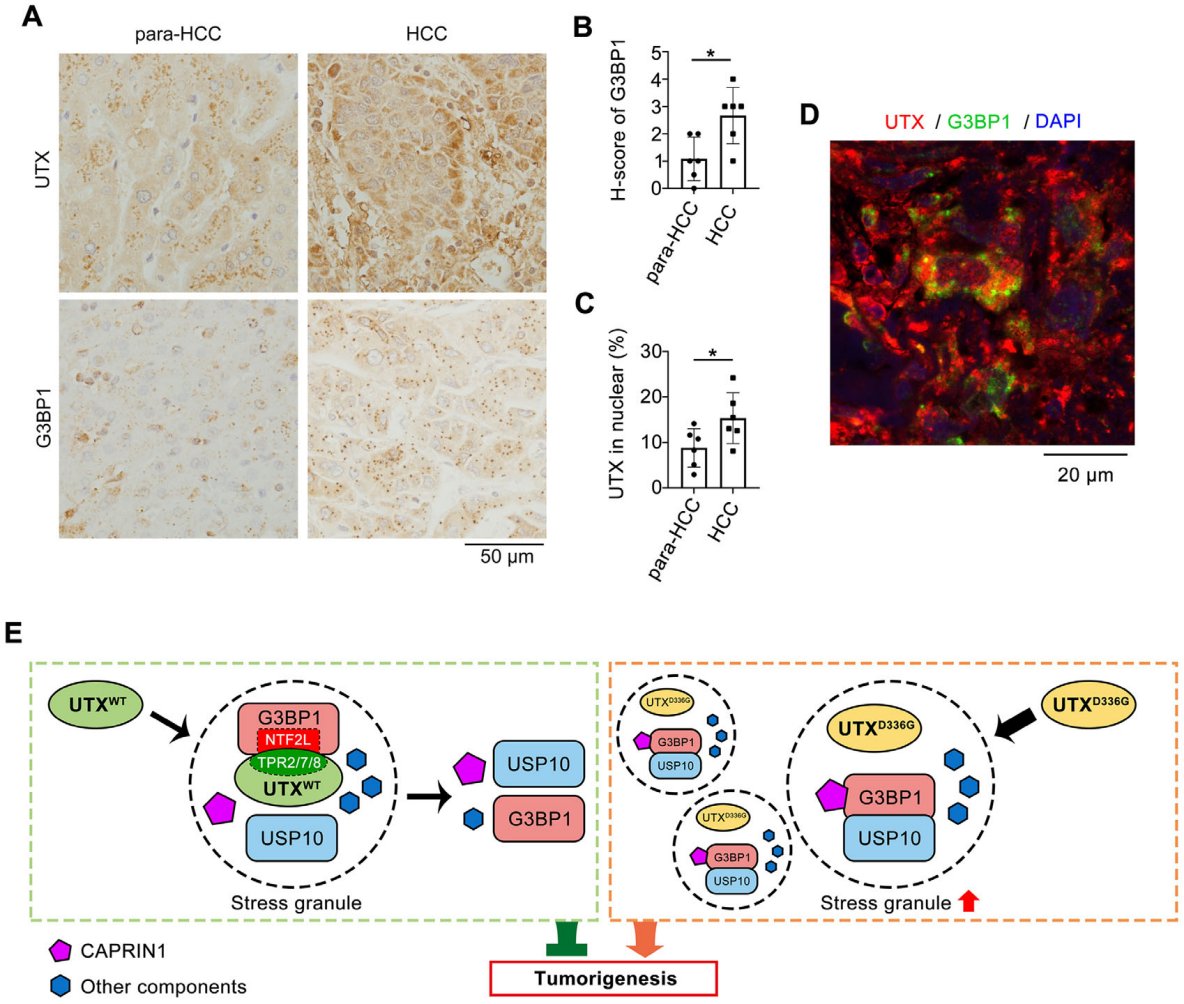
The association between UTX cytoplasmic localization and G3BP1 puncta in tumor tissues. A–C) Representative images of UTX and G3BP1 staining (A), with H‐score of G3BP1 (B) and percentage of cells with nuclear localization of UTX (C) in human HCC and para‐HCC sections. Scale bar, 50 µm. *n* = 6 per group. Data are shown as the mean ± SD, and analyzed by two‐tailed Student's *t*‐test. *p < 0.05. D) Representative images of UTX and G3BP1 staining in human lung cancer sections. UTX^+^, red; G3BP1^+^, green; DAPI stained nuclei, blue. Scale bar, 20 µm. E) A proposed model for how UTX and its clinical mutation are involved in tumorigenesis by affecting SG homeostasis.

To verify the association between UTX cytoplasmic localization and existing SGs in tumor tissues, immunohistochemical studies on human HCC sections were applied. G3BP1 staining showed puncta formation in both para‐HCC and HCC tissues; while compared with the para‐HCC parts, G3BP1 was significantly upregulated together with enhanced puncta formation in the HCC parts (**Figure** **9**A,B). Cytoplasmic localization of UTX was observed both in HCC and para‐HCC parts, whereas nuclear localization of UTX was slightly increased in HCC parts compared with para‐HCC parts (Figure 9A,C). Moreover, confocal results showed that cytoplasmic UTX co‐located with some SGs (G3BP1^+^ staining) in human lung cancer sections (Figure 9D).

## 3 Discussion

As a well‐known H3K27me3 demethylase, UTX plays pivotal roles in cellular and developmental processes such as embryogenesis, hematopoiesis, and cancer.^[^ ^21^ ^]^ A recent study indicated that UTX can phase separate in nuclei and regulates tumorigenesis.^[^ ^19^ ^]^ Here, we report that cytoplasmic UTX plays a novel role in regulating SGs under ER stress, and also plays important roles in tumorigenesis (Figure 9E). A recent study suggests that in COS‐7 cells (monkey kidney cells transformed by SV40), among all UTX domains, the IDR domain H3K27‐methylation‐activity dependently mediates UTX phase separation in the nuclei, while the TPR domain is critical for recruiting histone methyltransferase MLL4 to enrich nuclear H3K4 methylation levels; moreover, both domains contribute to the tumor suppressive function of UTX.^[^ ^19^ ^]^ Whereas in the present study, we identified a novel cytoplasmic role of UTX in tumor inhibition through its TPR domain, specifically the TPR motifs 2, 7, and 8, which binds G3BP1 to destabilize SGs (Figures 5 and 8). Interestingly, this effect neither depends on histone demethylase activity of UTX nor affects total cellular H3K4 methylation and DNA methylation levels (Figure S9, Supporting Information).

TPR‐containing proteins account for one‐third of mammalian protein chaperone network,^[^ ^40^ ^]^ which play important roles in cell proliferation,^[^ ^41^ ^]^ organelle targeting,^[^ ^42^ ^]^ and protein‐protein interactions.^[^ ^43^ ^]^ TPR consists of a 34‐residue helix‐turn‐helix motif that forms a repeating antiparallel α‐helical groove with an extended binding surface.^[^ ^44^ ^]^ The TPR domain of UTX has been reported to interact with several epigenetic factors, for example, it interacts with histone acetyltransferase CREB‐binding protein to regulate H3K27 acetylation in *Drosophila*.^[^ ^45^ ^]^ Here, we show that UTX regulates SG disassembly through the TPR domain by binding G3BP1 (Figures 4 and 5), which expands its interaction network and functions.

Recently, non‐classical UTX localization has been reported.^[^ ^25^ ^]^ Here, we found cytosolic localization of UTX is not incidental but plays physiological roles by regulating SG disassembly upon ER, heat, and oxidative stresses (Figures 1, 2, 3, 4; Figures S2–S4 and S8, Supporting Information). This function by the cytoplasmic UTX seems different from its gene transcriptional regulation function that mainly happened in nuclei, because the TPR domain of UTX regulates SGs disassembly without affects global H3K37me2/me3/ac, H3K4me3 and 5mC levels in cell lines we examined (Figure 4; Figure S9, Supporting Information). Whether the TPR domain of UTX plays roles in DNA methylation or histone methylation/acetylation on specific gene regulation that may contribute to its anti‐cancer effects, awaits further investigation.

SGs are critical for cells to adapt to various stresses, including ER stress, nutrient scarcity, and hypoxia.^[^ ^6^ ^]^ The existence of SGs is transient in healthy cells, however, mechanisms responsible for SG disassembly seems vary upon different stimulations and/or different stimulation durations. For examples, SG disassembly is regulated by autophagy under chronic oxidative stimulation such as sodium arsenite,^[^ ^46^ ^]^ while regulated by ubiquitination of G3BP1 under short‐term heat shock stimulation.^[^ ^27^ ^]^ Here, we proposed a new regulation on SG disassembly in which cytoplasmic UTX is recruited to the intra‐SG network hub, interacts with the NTF2L domain of G3BP1 and outcompetes G3BP1 binding partners to dissemble SGs (Figures 5 and 6).

The rapid growth of cancer cells heightens metabolism, alters tumor microenvironments, these persisting stresses may thus lead to SG formation. In different cancer types, SGs play complex roles. For examples, in HCC, a cancer type prone to ER and oxidative stress,^[^ ^47^ ^]^ SGs are suggested to play oncogenic roles in promoting cell proliferation, as well as enhancing drug resistance and immune escape.^[^ ^6^, ^48^, ^49^ ^]^ Whereas in pancreatic ductal adenocarcinoma (PDAC) with KRAS mutations, SGs are not found.^[^ ^50^ ^]^ A plausible explanation may due to KRAS mutated PDAC maintains strong basal autophagy to cope with various stresses, thus is less prone to ER stress.^[^ ^51^ ^]^ Therefore, the cancer‐promoting functions of SGs, as well as the cytoplasmic role of UTX in regulating SGs we reported, are likely tumor type or cancer stage dependent. This is might true when comparing UTX mutation rates in HCC and bladder cancer. For 14 HCC studies included in cBioPortal, 45 UTX mutations in 43 cases (2 samples harbor dual‐mutations) are found out of total 3951 cases, indicating a 1.1% UTX mutation rate. While, 151 UTX mutations in 139 cases (9 carry dual‐mutations, and 1 carry 4‐mutations) are identified in total 510 bladder cancer cases, indicating a 29.6% UTX mutation rate. Compared with bladder cancer, HCC showed higher mutation rates in TPR domain (35.5% vs 19.9%). Notably, most of UTX mutations are different in these two cancers, which may due to their differences in tumor initiation, major driver pathways, gene mutations, and metastatic organs.^[^ ^52^, ^53^ ^]^

The relationship between SG formation and cell proliferation have been studied, depletion of G3BP1 decreases cell proliferation and anchorage‐independent growth in malignant melanoma cells^[^ ^54^ ^]^; knockdown of G3BP1 inhibits cell proliferation by regulating β‐catenin signaling, which downregulates proliferation‐related genes in breast cancer cells.^[^ ^55^ ^]^ Consistently, we showed that G3BP1 KO cells reduced cell growth and abolished the effects of UTX in vitro and in vivo (Figure 8). Mechanisms by which SGs regulate cell proliferation have been suggested.^[^ ^6^ ^]^ G3BP1 may recruit cell cycle‐related mRNAs such as RNA binding fox‐1 homolog 2 to promote cancer cell proliferation,^[^ ^56^ ^]^ or may suppress peripheral myelin protein 22 levels to promote cancer cell proliferation.^[^ ^57^ ^]^

Cancer‐associated UTX mutations have been reported throughout its coding region, while their precise effects remain unclear. UTX mutations lacking TPR, JmjC, and/or IDR domain dramatically increase the level of DNA damage indicators RAD51 (DNA repair protein RAD51 homolog 1) and p‐γH2A.X, and cause cell apoptosis.^[^ ^58^ ^]^ Here, we found cancer‐associated UTX^D336G^ mutant promoted cell proliferation under ER stress (Figure 8A–D), affected UTX subcellular localization, promoted SGs formation (Figure 7). Furthermore, UTX^D336G^ promoted tumorigenesis, possibly by facilitating SG formation in nude mice (Figure 8E–G; Figure S12, Supporting Information). These results reveal a previously unknown function of a nuclear protein through its nonclassical subcellular localization.

Altogether, our findings indicate a new role of UTX, which enzyme activity independently regulates SG disassembly to assist in cellular responses to ER stress. These results unveil the physiological and pathological effects of UTX upon stresses and provide new angles for liver cancer development.

## 4 Experimental Section

### Cell Culture

HepG2, Huh7, and HEK293T cell lines were obtained from the China Center for Type Culture Collection (Wuhan, China); U2OS cell line was obtained from Procell Biotech. (Wuhan, China); STR was authenticated by the vendors, and mycoplasma contamination were tested routinely. Cells were cultured in DMEM (Hyclone, Logan, UT) supplemented with 10% FBS (Lonsera, Shanghai, China) and 1% penicillin–streptomycin (Hyclone), and maintained at 37 °C in a humidified incubator supplied with 5% CO_2_.

### Treatments

Thapsigargin (TG, 1–20 µm; Enzo Life Sci., Farmingdale, NY), sodium arsenite (NaAsO_2_, SA, 0.1–0.5 mm; Sigma–Aldrich, Saint Louis, MO) and DL‐Dithiothreitol (DTT, 1 mm; Beyotime, Shanghai, China) were dissolved following the manufacturers’ instructions and kept as stocking solutions at −20 °C until use. Working solutions were freshly prepared before use and cells were treated at indicated dosage and indicated time as shown in Figure legends. Cells were exposed to 43 °C for 1 h as heat shock stress.

### Isolation and Treatments of Primary Mouse Hepatocytes

Primary mouse hepatocytes were isolated as previously described.^[^ ^59^ ^]^ Briefly, mouse liver was perfused with calcium‐free solution, digested with type I collagenase (C0130, Sigma–Aldrich, St. Louis, MO), dispersed hepatocytes were collected and plated in collagen‐coated plates with DMEM media plus 10% FBS. Plates were washed 4 h later, and adherent hepatocytes were treated with DMSO or 10 µm TG for 1 h.

### Plasmid Constructs and Transfections

cDNA encoding human G3BP1 was obtained by PCR from HEK293T and/or U2OS cells using DNA polymerase (TaKaRa, Tokyo, Japan), which were subsequently used for subcloning. Flag‐tagged G3BP1 and its truncation variants were generated by cloning G3BP1 or truncates into a pRK‐5’Flag vector. CRISPR‐Cas9‐mediated modifications of U2OS cells were performed. Briefly, guide RNA (gRNA) targeting *G3BP1/G3BP2* (*G3BP1*: 5’‐TACCACACCATCATTTAGCG‐3’; *G3BP2*: 5’‐CAACGACCTAGAGAACGACC‐3’) were designed using CRISPOR (www.crispor.tefor.net/crispor.py) and synthesized oligonucleotides were ligated into LentiCRISPRv2 (Addgene, #52961) plasmid. G3BP1‐KO and G3BP2‐KO plasmids, or the control scramble plasmid, were packed into a lentivirus expression system, and stable knockout cell lines were selected by adding 2 µg mL^−1^ puromycin to the medium. Stable UTX knockout (gRNA targeting *UTX*: 5’‐AAAAGCGAGCGGCGAGAGCG‐3’) or G3BP1 knockout HepG2 cell lines were similarly generated, with stable knockout cell lines selected by 1 µg mL^−1^ puromycin.

Plasmids for human UTX (pFLAG‐UTX), shUTX and catalytic in‐activated UTX (an H1146A and E1148A double mutant) were kind gifts from Dr. Min Gyu Lee (MD Anderson Cancer Center). UTX truncated mutations (TPR deleted (UTX^△TPR^), IDR deleted (UTX^△IDR^), JmjC deleted (UTX^△JmjC^), or TPR only (UTX^TPR^), and pEGFP‐G3BP1 were obtained from MiaoLing Bio (Wuhan, China). UTX^D336G^ mutant was constructed by Tsingke Biotech (Wuhan, China). Plasmid for myc‐tagged UTX was constructed as previously reported.^[^ ^17^ ^]^ Flag‐tagged UTX TPR2, 5, 7, and 8 truncations were routinely constructed into a pRK‐5’Flag vector. Plasmids ddGA(GFPA)‐G3BP1, ddGB(GFPB)‐C1, and RFP‐TIA1 were constructed as previously reported.^[^ ^60^ ^]^ Plasmid ddGB‐UTX was constructed by subcloning the coding fragments of UTX into linearized ddGB‐C1 vector. Transient transfection was performed using Lipofectamine 2000 (Thermo Fisher, Waltham, MA).

### Mice and Tumor Xenograft

Male BALB/c nude mice were obtained from Hunan SJA Laboratory Animal Ltd. Mice were housed in ventilated microisolator cages with free access to water and food under a 12:12 h light/dark cycle in a specific pathogen‐free, temperature‐controlled room (22 ± 2 °C). Animals were handled according to the Guidelines of China Animal Welfare Legislation, and approved by the Committee on Ethics in the Care and Use of Laboratory Animals of the College of Life Sciences, Wuhan University (WDSKY0201705).

Both flanks of nude mice (6 weeks old) were subcutaneously injected with 1 × 10^7^ WT or G3BP1 KO HepG2 cells transfected with either empty vector pCMV, or UTX^WT^ or UTX^TPR^ or UTX^D336G^ and counted as day 0. Mice were killed at day 4 or day 10, xenograft tumors were dissected and weighed, and volumes were calculated as described.^[^ ^59^, ^61^ ^]^

### Dot Blot Assays

Genomic DNA was isolated using a genomic DNA extraction kit (Tiangen Biotech., Beijing, China), and dot blot assays were performed.^[^ ^62^, ^63^ ^]^ Briefly, 100 or 200 ng of denatured DNA was spotted onto a nitrocellulose membrane (Bio‐Rad, Hercules, CA). After cross‐linking and blocking, anti‐5mC antibody was applied, then probed with HRP‐conjugated secondary antibody (Bio‐Rad), visualized by enhanced chemiluminescence reagent (Beyotime), and quantitated using Quantity One (Bio‐Rad).

### Prediction of Protein‐Protein Interactions

Interaction between the TPR domain of UTX and G3BP1 was modeled by AlphaFold2 using ColabFold (https://colab.research.google.com/ssssub/sokrypton/ColabFold) and interaction interfaces were further analyzed using PDBePISA (www.ebi.ac.uk/msd‐srv/prot_int/pistart.html) as previously reported.^[^ ^64^, ^65^ ^]^ The binding affinity for the TPR domain of UTX and G3BP1 were analyzed by Δ^i^G (solvation energies), protein‐protein interaction was visualized with Pymol 2.3.0 as previously reported.^[^ ^66^ ^]^ Furthermore, interaction network of G3BP1 was predicted by the STRING (https://string‐db.org).

### Expression and Purification of Recombinant Proteins

cDNAs encoding human UTX TPR domain and G3BP1 NTF2L domain were cloned into pET30c‐6×his vector (primers listed in Table S1, Supporting Information). Their expression and purification were conducted as previously described with modifications.^[^ ^67^ ^]^ Briefly, plasmids expressing these proteins were transformed into *E. coli* BL21 (DE3), and expression was induced. Collected cells were harvested and resuspended in wash buffer (20 mm Tris‐HCl pH 8.0, 10 mm EDTA, 1% Triton X‐100, 1 mm PMSF). Following sonication, the inclusion bodies were collected and dissolved in lysis buffer (50 mm CAPS pH 11.0, 0.3% sarcosyl, 1 mm DTT) at 4 °C overnight, and the supernatant was dialyzed to refold (20 mm Tris‐HCl pH 8.0, 1 mm DTT) the recombinant TPR or NTF2L proteins.

### Microscale Thermophoresis (MST) Analysis

Microscale thermophoresis (MST) was conducted as previously reported.^[^ ^68^ ^]^ In brief, the binding affinity of G3BP1‐NTF2L against UTX‐TPR was measured using Monolith NT.115 (NanoTemper Tech., Munich, Germany). FAM‐labeled proteins were stored at 100 nmol L^−1^ in MST buffer (20 mmol L^−1^ Tris, pH 8.0, 0.05% Tween 20). Labeled proteins were mixed with unlabeled peptides in 16 serially diluted concentrations and incubated for 10 min at 25 °C in the darkness, then loaded into premium capillaries (NanoTemper Tech.) and measured at 25 °C with 20% LED power and medium MST power. Experiments were repeated three times, data were analyzed using MO. Affinity Analysis Software, version 2.3 (NanoTemper Tech.).

### Co‐Immunoprecipitation (Co‐IP)

Co‐IP was carried out as previously described.^[^ ^69^ ^]^ Briefly, cells were lysed with IP buffer (Beyotime), and cell lysates were incubated with specific antibodies (Table S3, Supporting Information) or respective IgGs with Dynabeads Protein G (Thermo Fisher) overnight at 4 °C. After washing (pre‐lysis buffer with additional 50 mm NaCl), the beads were boiled in loading buffer and subjected to immunoblotting.

### Human HCC and Lung Cancer Specimens

HCC and para‐HCC paraffin sections from six patients, and lung cancer paraffin sections from four patients were obtained from the Affiliated Hubei Cancer Hospital (Wuhan, China) and the Affiliated Tongji Hospital, Tongji Medical College (Wuhan, China), respectively. Collection and use of patient paraffin sections were approved by the institutional review boards of the hospitals (LHBCH2022YN‐015 and TJ‐IRB202408023, respectively), all subjects included in the study provided informed consent. All research was conducted in accordance with both the Declarations of Helsinki and Istanbul.

### Immunofluorescence Staining and Immunohistochemical Staining

Immunofluorescence staining was carried out as previously described.^[^ ^16^ ^]^ Briefly, cells were grown on glass coverslips (NEST, Wuxi, China) followed by transfection and/or treatments as indicated. Cryosections of xenograft samples were obtained routinely. Cells or cryosections of xenograft samples were incubated with indicated primary antibodies (Table S3, Supporting Information), followed by the respective secondary antibodies (all from Invitrogen, Carlsbad, CA). Nuclei were stained with 4’,6‐diamidino‐2‐phenylindole (DAPI, Sigma–Aldrich). Images for fixed cells or xenograft cryosections were captured using a TCS SP8 confocal microscope (Leica, Germany) with a 63× objective.

Paraffin‐embedded sections of human liver and lung cancer samples were deparaffinized and rehydrated as previously reported.^[^ ^70^ ^]^ Liver cancer sections were incubated with 3% H_2_O_2_ to quench endogenous peroxidase activity. After blocking, primary antibodies including UTX or G3BP1 were applied (detailed information in Table S3, Supporting Information). After washing, sections were incubated with biotinylated anti‐rabbit secondary antibody and ABC‐peroxidase solution (Vector Laboratories, Burlingame, CA) sequentially. Positive staining was visualized using DAB substrate following the protocol (Vector laboratories). Sample pictures were taken using an Olympus BX51 microscope and analyzed using Histo‐score as reported.^[^ ^47^ ^]^ While, lung cancer sections were antigens retrieved by microwaving slides in Tris‐EDTA buffer (pH 9.0). Subsequently, sections incubated overnight at 4 °C with primary antibodies UTX and G3BP1, combined with multiplex‐fluorescence immunohistochemical mouse/rabbit kit (Immunoway, Los Angels, CA) according to the manufacturer's manual, and immunofluorescence images were captured and imaged using a TCS SP8 confocal microscope.

For living cell imaging, stress granules were acquired with a LSM 980 (Zeiss, Germany) equipped with Airyscan2 detectors and 63×/1.4‐NA plan Apochromat oil objective within a chamber at 37 °C with 5% CO_2_. Super‐resolution images of stress granules and UTX puncta were obtained using the HIS‐SIM (High Sensitivity Structured Illumination Microscope, Guangzhou Computational Super‐Resolution Biotech., Guangzhou, China), by a 100× Plan‐Apochromat oil objective lens, 1.7‐NA. Images of cells or sections from different conditions were captured under identical conditions with respect to laser intensity and exposure, and processed with LAS X or Zeiss ZEN blue software. For stress granules quantification, images containing SGs were transposed into 8‐bit images, a “RenyiEntropy‐automated thresholding” method in Image J (National Institutes of Health) providing the best SGs coverage was applied. Cells with two or more cytoplasmic G3BP1^+^ puncta were defined as SG‐positive cells. The number of SGs were manually counted, and the size of SGs were analyzed using the “analyze particles” plug‐in in Image J.

### Subcellular Fractionation

Subcellular fractionation was performed at 4 °C as previously reported.^[^ ^60^ ^]^ Briefly, cells were lysed by adding 1 mL buffer L (pH 7.6, 50 mm Tris‐HCl, 50 mm NaCl, 5 mm MgCl_2_, 0.1% NP‐40, 1 mm β‐mercaptoethanol, EDTA‐free protease inhibitor cocktail and phosphatase inhibitor). After lysis for 30 min on a rotary shaker, whole cell lysates were subjected to centrifugation at 2000 g for 5 min to remove nuclei. Supernatants were further centrifuged at 10 000 g for 10 min. The pellets were collected as the ribonucleoprotein granule fraction after washing with buffer L for three times. The subcellular fractions were boiled in protein loading buffer for immunoblots.

### MTT Assays

Cells were transiently transfected with indicated plasmids and then stimulated with indicated treatments, MTT assays were carried out as previously described.^[^ ^59^ ^]^

### EdU Assays

Cells were seeded in 12‐well plates and maintained for 24 h before transfected with indicated plasmids. EdU staining was performed using a EdU Cell Proliferation Kit (Beyotime) following the manufacturer's instruction. Images from three random fields were taken, and the percentage of EdU‐positive cells was calculated.

### Quantitative Real‐Time PCR (qPCR) and Western Blots

qPCR and Western blots were performed as previously described.^[^ ^16^ ^]^ Primers and primary antibodies used in the present study are provided in Tables S2 and S3 (Supporting Information). *Actb* was used as the qPCR internal control. Targeted protein bands were quantitated to the respective loading control in the same sample by Quantity One (Bio‐Rad).

### Statistical Analysis

Most of the data were not pre‐processed, except for the quantifications of WB, co‐IP, qPCR, or dot blots analysis, the levels of target proteins or genes or 5mC (molecule of interest) were first quantitated relative to the internal control (if applicable) in the same sample, then they were further normalized to the respective control group, which was arbitrarily set as one‐fold. All results were presented as the mean ± SD (standard deviation). n number for in vivo studies and the number of biological repeats for in vitro studies are provided in the corresponding figure legends. All the cell experiments were repeated at least three times, and *n* ≥ 4 for animal studies. Statistical analysis was performed using two‐tailed Student's *t*‐test for two experimental groups, and one‐way ANOVA for multiple experimental groups without adjustment. All statistical analyses were performed using GraphPad Prism 9.0. Differences were considered statistically significant at *p < 0.05, **p < 0.01, ***p < 0.001.

## Conflict of Interest

The authors declare no conflict of interest.

## Author Contributions

X.K.L., L.Z., and K.H. designed the study and analyzed the data. X.K.L., M.X., Y.X., R.Q., D.W., Y.Z., J.W., Y.Y., L.Y., T.S., H.S., X.R.L., H.C., performed the experiments. Y.K.L. and Y.L. provided materials. X.K.L., X.R.L., Y.C., L.Z., and K.H. wrote the manuscript.

## Supporting information



Supporting Information

## Data Availability

The data that support the findings of this study are available from the corresponding author upon reasonable request.

## References

[1] J. Y. Youn , B. J. A. Dyakov , J. Zhang , J. D. R. Knight , R. M. Vernon , J. D. Forman‐Kay , A. C. Gingras , Mol. Cell 2019, 76, 286. 31626750 10.1016/j.molcel.2019.09.014

[2] D. Tauber , G. Tauber , R. Parker , Trends Biochem. Sci. 2020, 45, 764. 32475683 10.1016/j.tibs.2020.05.002 PMC7211619

[3] P. Yang , C. Mathieu , R. M. Kolaitis , P. Zhang , J. Messing , U. Yurtsever , Z. Yang , J. Wu , Y. Li , Q. Pan , J. Yu , E. W. Martin , T. Mittag , H. J. Kim , J. P. Taylor , Cell 2020, 181, 325. 32302571 10.1016/j.cell.2020.03.046 PMC7448383

[4] N. Kedersha , P. Ivanov , P. Anderson , Trends Biochem. Sci. 2013, 38, 494. 24029419 10.1016/j.tibs.2013.07.004 PMC3832949

[5] D. Fujikawa , T. Nakamura , D. Yoshioka , Z. Li , H. Moriizumi , M. Taguchi , N. Tokai‐Nishizumi , H. Kozuka‐Hata , M. Oyama , M. Takekawa , Curr. Biol. 2023, 33, 1967. 37119817 10.1016/j.cub.2023.04.012

[6] H. Zhou , J. Luo , K. Mou , L. Peng , X. Li , Y. Lei , J. Wang , S. Lin , Y. Luo , L. Xiang , Cell Biosci. 2023, 13, 86. 37179344 10.1186/s13578-023-01030-6 PMC10182661

[7] G. Cereghetti , C. Wilson‐Zbinden , V. M. Kissling , M. Diether , A. Arm , H. Yoo , I. Piazza , S. Saad , P. Picotti , D. A. Drummond , U. Sauer , R. Dechant , M. Peter , Nat. Cell Biol. 2021, 23, 1085. 34616026 10.1038/s41556-021-00760-4 PMC7611853

[8] M. Fang , Y. Liu , C. Huang , S. Fan , BioFactors 2024, 50, 422. 37966813 10.1002/biof.2017

[9] S. Hong , Y. W. Cho , L. R. Yu , H. Yu , T. D. Veenstra , K. Ge , Proc. Natl. Acad. Sci. USA 2007, 104, 18439. 18003914 10.1073/pnas.0707292104 PMC2141795

[10] J. K. Wang , M. C. Tsai , G. Poulin , A. S. Adler , S. Chen , H. Liu , Y. Shi , H. Y. Chang , Genes Dev. 2010, 24, 327. 20123895 10.1101/gad.1882610 PMC2816731

[11] H. Faralli , C. Wang , K. Nakka , A. Benyoucef , S. Sebastian , L. Zhuang , A. Chu , C. G. Palii , C. Liu , B. Camellato , M. Brand , K. Ge , F. J. Dilworth , J. Clin. Invest. 2016, 126, 1555. 26999603 10.1172/JCI83239 PMC4811158

[12] K. B. Shpargel , T. Sengoku , S. Yokoyama , T. Magnuson , PLoS Genet. 2012, 8, 1002964. 10.1371/journal.pgen.1002964 PMC3459986 23028370

[13] S. Lee , J. W. Lee , S. K. Lee , Dev. Cell 2012, 22, 25. 22192413 10.1016/j.devcel.2011.11.009 PMC4111644

[14] X. Lei , J. Jiao , Stem Cell Rep. 2018, 10, 1193. 10.1016/j.stemcr.2018.02.008 PMC5998300 29551674

[15] Y. Huang , Y. Xie , D. Yang , M. Xiong , X. Chen , D. Wu , Q. Wang , H. Chen , L. Zheng , K. Huang , Pharmacol. Res. 2022, 175, 106021. 34883214 10.1016/j.phrs.2021.106021

[16] H. Chen , C. Liu , Q. Wang , M. Xiong , X. Zeng , D. Yang , Y. Xie , H. Su , Y. Zhang , Y. Huang , Y. Chen , J. Yue , C. Liu , S. Wang , K. Huang , L. Zheng , Nat. Commun. 2022, 13, 3835. 35788583 10.1038/s41467-022-31476-0 PMC9253056

[17] H. Chen , Y. Huang , X. Zhu , C. Liu , Y. Yuan , H. Su , C. Zhang , C. Liu , M. Xiong , Y. Qu , P. Yun , L. Zheng , K. Huang , J. Physiol. 2019, 597, 1643. 30516825 10.1113/JP277367 PMC6418754

[18] S. Majumder , K. Thieme , S. N. Batchu , T. A. Alghamdi , B. B. Bowskill , M. G. Kabir , Y. Liu , S. L. Advani , K. E. White , L. Geldenhuys , K. K. Tennankore , P. Poyah , F. S. Siddiqi , A. Advani , J. Clin. Invest. 2018, 128, 483. 29227285 10.1172/JCI95946 PMC5749498

[19] B. Shi , W. Li , Y. Song , Z. Wang , R. Ju , A. Ulman , J. Hu , F. Palomba , Y. Zhao , J. P. Le , W. Jarrard , D. Dimoff , M. A. Digman , E. Gratton , C. Zang , H. Jiang , Nature 2021, 597, 726. 34526716 10.1038/s41586-021-03903-7 PMC9008583

[20] Z. G. Xiao , J. Shen , L. Zhang , L. F. Li , M. X. Li , W. Hu , Z. J. Li , C. H. Cho , Curr. Med. Chem. 2016, 23, 3687. 27458035 10.2174/0929867323666160725093522

[21] N. Tran , A. Broun , K. Ge , Mol. Cell. Biol. 2020, 40, 00341. 10.1128/MCB.00341-20 PMC7523656 32817139

[22] I. Gazova , A. Lengeling , K. M. Summers , Mol. Genet. Metab. 2019, 127, 31. 31097364 10.1016/j.ymgme.2019.04.012

[23] R. Wiedemuth , S. Thieme , K. Navratiel , B. Dorschner , S. Brenner , Int. J. Biochem. Cell Biol. 2018, 97, 78. 29421189 10.1016/j.biocel.2018.02.004

[24] A. Lang , M. Yilmaz , C. Hader , S. Murday , X. Kunz , N. Wagner , C. Wiek , P. Petzsch , K. Köhrer , J. Koch , M. J. Hoffmann , A. Greife , W. A. Schulz , Cancers 2019, 11, 11040481. 10.3390/cancers11040481 PMC6520694 30987376

[25] H. Kato , K. Asamitsu , W. Sun , S. Kitajima , N. Yoshizawa‐Sugata , T. Okamoto , H. Masai , L. Poellinger , Oncogene 2020, 39, 3322. 32071397 10.1038/s41388-020-1218-3

[26] J. R. Child , Q. Chen , D. W. Reid , S. Jagannathan , C. V. Nicchitta , RNA 2021, 27, 1241. 34244458 10.1261/rna.078858.121 PMC8456999

[27] Y. Gwon , B. A. Maxwell , R. M. Kolaitis , P. Zhang , H. J. Kim , J. P. Taylor , Science 2021, 372, abf6548. 10.1126/science.abf6548 PMC8574224 34739333

[28] B. C. Cui , V. Sikirzhytski , M. Aksenova , M. D. Lucius , G. H. Levon , Z. T. Mack , C. Pollack , D. Odhiambo , E. Broude , S. B. Lizarraga , M. D. Wyatt , M. Shtutman , Biochem. Pharmacol. 2020, 182, 114280. 33049245 10.1016/j.bcp.2020.114280 PMC7686075

[29] S. Namkoong , A. Ho , Y. M. Woo , H. Kwak , J. H. Lee , Mol. Cell 2018, 70, 175. 29576526 10.1016/j.molcel.2018.02.025 PMC6359928

[30] S. Jain , J. R. Wheeler , R. W. Walters , A. Agrawal , A. Barsic , R. Parker , Cell 2016, 164, 487. 26777405 10.1016/j.cell.2015.12.038 PMC4733397

[31] S. C. Alford , Y. Ding , T. Simmen , R. E. Campbell , ACS Synth. Biol. 2012, 1, 569. 23656278 10.1021/sb300050j PMC3653836

[32] K. Arimoto‐Matsuzaki , H. Saito , M. Takekawa , Nat. Commun. 2016, 7, 10252. 26738979 10.1038/ncomms10252 PMC4729832

[33] J. Van der Meulen , F. Speleman , P. Van Vlierberghe , Epigenetics 2014, 9, 658. 24561908 10.4161/epi.28298 PMC4063824

[34] J. H. Kim , A. Sharma , S. S. Dhar , S. H. Lee , B. Gu , C. H. Chan , H. K. Lin , M. G. Lee , Cancer Res. 2014, 74, 1705. 24491801 10.1158/0008-5472.CAN-13-1896 PMC3962500

[35] X. Wang , W. Rosikiewicz , Y. Sedkov , T. Martinez , B. S. Hansen , P. Schreiner , J. Christensen , B. Xu , S. M. Pruett‐Miller , K. Helin , H. M. Herz , Life Sci. Alliance 2022, 5, 2101228. 10.26508/lsa.202101228 PMC8548262 34667079

[36] H. Sidibe , A. Dubinski , C. Vande Velde , J. Neurochem. 2021, 157, 944. 33349931 10.1111/jnc.15280 PMC8248322

[37] D. Song , L. Kuang , L. Yang , L. Wang , H. Li , X. Li , Z. Zhu , C. Shi , H. Zhu , W. Gong , Proc. Natl. Acad. Sci. USA 2022, 119, 2207975119. 10.1073/pnas.2207975119 PMC9636964 36279435

[38] D. W. Sanders , N. Kedersha , D. S. W. Lee , A. R. Strom , V. Drake , J. A. Riback , D. Bracha , J. M. Eeftens , A. Iwanicki , A. Wang , M. T. Wei , G. Whitney , S. M. Lyons , P. Anderson , W. M. Jacobs , P. Ivanov , C. P. Brangwynne , Cell 2020, 181, 306. 32302570 10.1016/j.cell.2020.03.050 PMC7816278

[39] N. Kedersha , M. D. Panas , C. A. Achorn , S. Lyons , S. Tisdale , T. Hickman , M. Thomas , J. Lieberman , G. M. McInerney , P. Ivanov , P. Anderson , J. Cell Biol. 2016, 212, 845. 27022092 10.1083/jcb.201508028 PMC4810302

[40] M. Brehme , C. Voisine , T. Rolland , S. Wachi , J. H. Soper , Y. Zhu , K. Orton , A. Villella , D. Garza , M. Vidal , H. Ge , R. I. Morimoto , Cell Rep. 2014, 9, 1135. 25437566 10.1016/j.celrep.2014.09.042 PMC4255334

[41] M. Tufano , L. Marrone , C. D'Ambrosio , V. Di Giacomo , S. Urzini , Y. Xiao , M. Matuozzo , A. Scaloni , M. F. Romano , S. Romano , Cell Death Dis. 2023, 14, 116. 36781840 10.1038/s41419-023-05629-y PMC9925821

[42] G. Muthukumar , T. A. Stevens , A. J. Inglis , T. K. Esantsi , R. A. Saunders , F. Schulte , R. M. Voorhees , A. Guna , J. S. Weissman , Mol. Cell 2024, 84, 1101. 38428433 10.1016/j.molcel.2024.01.028

[43] N. Zeytuni , R. Zarivach , Structure 2012, 20, 397. 22404999 10.1016/j.str.2012.01.006

[44] L. D. D'Andrea , L. Regan , Trends Biochem. Sci. 2003, 28, 655. 14659697 10.1016/j.tibs.2003.10.007

[45] F. Tie , R. Banerjee , P. A. Conrad , P. C. Scacheri , P. J. Harte , Mol. Cell. Biol. 2012, 32, 2323. 22493065 10.1128/MCB.06392-11 PMC3372260

[46] J. R. Buchan , R. M. Kolaitis , J. P. Taylor , R. Parker , Cell 2013, 153, 1461. 23791177 10.1016/j.cell.2013.05.037 PMC3760148

[47] X. Liu , Q. Ma , Z. Jia , Y. Zhou , C. Zou , Y. Xiao , Y. Chen , C. Ma , L. Song , J. Yang , C. Wang , H. Xu , H. Chen , J. Shi , J. Yue , Y. Sun , D. Hu , R. B. Petersen , Y. Li , A. Peng , K. Huang , L. Zheng , Adv. Sci. 2025, 12, 2416401. 10.1002/advs.202416401 PMC12097128 40126377

[48] Y. Jia , R. Jia , Z. Dai , J. Zhou , J. Ruan , W. Chng , Z. Cai , X. Zhang , iScience 2024, 27, 110359. 39100690 10.1016/j.isci.2024.110359 PMC11295550

[49] M. Lavalée , N. Curdy , C. Laurent , J. J. Fournié , D. M. Franchini , Trends Cancer 2021, 7, 902. 34144941 10.1016/j.trecan.2021.05.006

[50] M. Libert , S. Quiquempoix , J. S. Fain , S. Pyr Dit Ruys , M. Haidar , M. Wulleman , G. Herinckx , D. Vertommen , C. Bouchart , T. Arsenijevic , J. L. Van Laethem , P. Jacquemin , EMBO Rep. 2024, 25, 4693. 39390257 10.1038/s44319-024-00284-6 PMC11549491

[51] M. F. Montenegro , R. Martí‐Díaz , A. Navarro , J. Tolivia , L. Sánchez‐Del‐Campo , J. Cabezas‐Herrera , J. N. Rodríguez‐López , Cell Death Dis. 2023, 14, 761. 37996408 10.1038/s41419-023-06288-9 PMC10667277

[52] S. Faivre , L. Rimassa , R. S. Finn , J. Hepatol. 2020, 72, 342. 31954496 10.1016/j.jhep.2019.09.010

[53] M. Olislagers , F. C. de Jong , V. C. Rutten , J. L. Boormans , T. Mahmoudi , T. C. M. Zuiverloon , Nat. Rev. Urol. 2025, 22, 75. 39095581 10.1038/s41585-024-00914-7

[54] N. Oi , J. Yuan , M. Malakhova , K. Luo , Y. Li , J. Ryu , L. Zhang , A. M. Bode , Z. Xu , Y. Li , Z. Lou , Z. Dong , Oncogene 2015, 34, 2660. 24998844 10.1038/onc.2014.194 PMC4286533

[55] C. H. Zhang , H. Liu , W. L. Zhao , W. X. Zhao , H. M. Zhou , R. G. Shao , Acta Pharmacol. Sin. 2021, 42, 1900. 33536604 10.1038/s41401-020-00598-w PMC8563869

[56] S. Choi , M. Sa , N. Cho , K. K. Kim , S. H. Park , Exp. Mol. Med. 2019, 51, 1. 10.1038/s12276-019-0246-y PMC6486603 31028247

[57] S. Winslow , K. Leandersson , C. Larsson , Mol. Cancer 2013, 12, 156. 24321297 10.1186/1476-4598-12-156 PMC3866477

[58] J. Koch , A. Lang , P. Whongsiri , W. A. Schulz , M. J. Hoffmann , A. Greife , BMC Mol. Cell Biol. 2021, 22, 54. 34702163 10.1186/s12860-021-00394-2 PMC8549169

[59] Q. Wang , Y. Chen , Y. Xie , D. Yang , Y. Sun , Y. Yuan , H. Chen , Y. Zhang , K. Huang , L. Zheng , Cancer Sci. 2022, 113, 1679. 35294987 10.1111/cas.15336 PMC9128180

[60] S. Liu , X. Zhang , X. Yao , G. Wang , S. Huang , P. Chen , M. Tang , J. Cai , Z. Wu , Y. Zhang , R. Xu , K. Liu , K. He , Y. Wang , L. Jiang , Q. A. Wang , L. Rui , J. Liu , Y. Liu , Nat. Cell Biol. 2024, 26, 917. 38714852 10.1038/s41556-024-01418-7

[61] Y. Sun , Q. Wang , Y. Zhang , M. Geng , Y. Wei , Y. Liu , S. Liu , R. B. Petersen , J. Yue , K. Huang , L. Zheng , J. Hepatol. 2020, 73, 603. 32593682 10.1016/j.jhep.2020.03.050

[62] Y. Yuan , C. Liu , X. Chen , Y. Sun , M. Xiong , Y. Fan , R. B. Petersen , H. Chen , K. Huang , L. Zheng , Mol. Nutr. Food Res. 2021, 65, 2100417. 10.1002/mnfr.202100417 34129274

[63] Y. Fan , Y. Yuan , M. Xiong , M. Jin , D. Zhang , D. Yang , C. Liu , R. B. Petersen , K. Huang , A. Peng , L. Zheng , Theranostics 2023, 13, 5348. 37908721 10.7150/thno.87416 PMC10614682

[64] X. Li , L. Yu , X. Liu , T. Shi , Y. Zhang , Y. Xiao , C. Wang , L. Song , N. Li , X. Liu , Y. Chen , R. B. Petersen , X. Cheng , W. Xue , Y. V. Yu , L. Xu , L. Zheng , H. Chen , K. Huang , Nat. Commun. 2024, 15, 8748. 39384788 10.1038/s41467-024-53086-8 PMC11464764

[65] L. Yu , X. Li , T. Shi , N. Li , D. Zhang , X. Liu , Y. Xiao , X. Liu , R. B. Petersen , W. Xue , Y. V. Yu , D. S. Hu , L. Xu , H. Chen , L. Zheng , K. Huang , A. Peng , Int. J. Biol. Macromol. 2025, 297, 139875. 39818366 10.1016/j.ijbiomac.2025.139875

[66] C. Yang , H. Xu , D. Yang , Y. Xie , M. Xiong , Y. Fan , X. Liu , Y. Zhang , Y. Xiao , Y. Chen , Y. Zhou , L. Song , C. Wang , A. Peng , R. B. Petersen , H. Chen , K. Huang , L. Zheng , Nat. Commun. 2023, 14, 4261. 37460623 10.1038/s41467-023-40036-z PMC10352345

[67] J. Yang , S. Y. Wan , Q. Y. Song , Y. H. Xie , J. Wan , Y. H. Zhou , Z. T. Zhang , Y. S. Xiao , X. Li , H. Chen , X. R. Liu , L. Xu , H. J. You , D. S. Hu , R. B. Petersen , Y. H. Zhang , L. Zheng , Y. Zhang , K. Huang , Cell Death Differ. 2025, 32, 672. 39592710 10.1038/s41418-024-01422-2 PMC11982567

[68] Y. Xiao , Z. Tong , H. Xu , Z. Jia , C. Wang , Y. Cao , L. Song , S. Hao , J. Yang , Y. Zhou , Y. Xie , P. Wu , T. He , Y. Wu , R. B. Petersen , A. Peng , C. Zhang , H. Chen , L. Zheng , K. Huang , Sci. Adv. 2025, 11, adt3943. 10.1126/sciadv.adt3943 PMC12047437 40315322

[69] D. Yang , Y. Fan , M. Xiong , Y. Chen , Y. Zhou , X. Liu , Y. Yuan , Q. Wang , Y. Zhang , R. B. Petersen , H. Su , J. Yue , C. Zhang , H. Chen , K. Huang , L. Zheng , EMBO Rep. 2023, 24, 56128. 10.15252/embr.202256128 PMC10240209 37042626

[70] Y. Chen , J. Shi , X. Wang , L. Zhou , Q. Wang , Y. Xie , C. Peng , L. Kuang , D. Yang , J. Yang , C. Yang , X. Li , Y. Yuan , Y. Zhou , A. Peng , Y. Zhang , H. Chen , X. Liu , L. Zheng , K. Huang , Y. Li , Proc. Natl. Acad. Sci. USA 2023, 120, 2306288120. 10.1073/pnas.2306288120 PMC10523483 37729198

