# Supplementary material for: Histone Demethylase UTX Suppresses Tumor Cell Proliferation by Regulating Stress Granules
Source: Adv Sci (Weinh). 2025 Jun 19;12(34):e01990. doi: 10.1002/advs.202501990 (PMC12442641; doi:10.1002/advs.202501990)
Supplement: Supplementary file 1 — Supporting Information [file ADVS-12-e01990-s001.doc]

**Supporting Information**

*for*

**Histone Demethylase UTX Suppresses Tumor Cell Proliferation by Regulating Stress Granules**

Xikai Liu^1^, Xinran Liu^2^, Mei Xue^1^, Yushuo Xiao^2^, Yuchen Chen^2^, Rong Qiu^1^, Di Wu^1^, Yihao Zhou^1^, Jiao Wang^3^, Yan Yuan^1^, Linwei Yu^2^, Tianyi Shi^2^, Yangkai Li^4^, Hua Su^5^, Hong Chen^2^, Yong Liu^1^, Kun Huang^2^*, Ling Zheng^1^*

^1^State Key Laboratory of Metabolism and Regulation in Complex Organisms, Hubei Key Laboratory of Cell Homeostasis, TaiKang Center for Life and Medical Sciences; Frontier Science Center for Immunology and Metabolism, College of Life Sciences, Wuhan University, Wuhan, China, 430072

^2^School of Pharmacy, Tongji Medical College and State Key Laboratory for Diagnosis and Treatment of Severe Zoonotic Infectious Diseases, Huazhong University of Science and Technology, Wuhan, China, 430030

^3^Wuhan No.1 Hospital, Tongji Medical College, Huazhong University of Science and Technology, Wuhan, China, 430000

^4^Department of Thoracic Surgery, Tongji Hospital, Tongji Medical College, Huazhong University of Science and Technology, Wuhan, China, 430030

^5^Department of Nephrology, Union Hospital, Tongji Medical College, Huazhong University of Science and Technology, Wuhan, China, 430022

*Corresponding Authors*

Kun Huang, Ph.D., Tongji School of Pharmacy, Huazhong University of Science & Technology, Wuhan, China, 430030, [kunhuang@hust.edu.cn](mailto:kunhuang@hust.edu.cn)

Ling Zheng, Ph.D., College of Life Sciences, Wuhan University, Wuhan, China 430072, [lzheng@whu.edu.cn](mailto:lzheng@whu.edu.cn)

This file includes: Supplemental Tables S1 to S4, and Figures S1 to S13.

**Supplemental Table 1**. **Primers for plasmids constructed in the study.**

| **Primer name** | **Sequence (5’-3’)** | |
| --- | --- | --- |
| G3BP1-WT | | Forward ATAGTCGACAATGGTGATGGAGAAGCCTAGTCCC  Reverse ATAGCGGCCGCTCACTGCCGTGGCGCAAGCCC |
| G3BP1-NTF2L | | Forward ATAGTCGACAATGGATGAGGTCTTTGGTGGGTTTGTC  Reverse ATAGCGGCCGCTCAATCTTGGTATCTGAAGATATCATTG |
| G3BP1-Δ(RRM+RGG) | | Forward ATAGTCGACAATGAGTCACCAACTCTTCATTGGCAACC  Reverse ATAGCGGCCGCTCAACTGTCAGGGTGTCTCACCATTC |
| G3BP1-ΔNTF2L | | Forward ATAGCGGCCGCTCAATCTTGGTATCTGAAGATATCATTG  Reverse ATAGCGGCCGCTCACTGCCGTGGCGCAAGCCC |
| G3BP1-RRM+RGG | | Forward ATAGCGGCCGCTCAACTGTCAGGGTGTCTCACCATTC  Reverse ATAGCGGCCGCTCACTGCCGTGGCGCAAGCCC |
| pRK-TPR | | Forward ATAGTCGACAATGAAATCCTGCGGAGTGTCGCTCG  Reverse ATAGCGGCCGCTCAAGAGACGCTAGGCACTCTGGTCA |
| pRK-ΔTPR2 | | Forward TCTGACTACTGGAAGAATCATTTACGACTTGGGCTTA  Reverse TAAGCCCAAGTCGTAAATGATTCTTCCAGTAGTCAGA |
| pRK-ΔTPR5 | | Forward ACTGTCTTACAACAGTTAGGCCAGTCCTGGTATTT  Reverse AAATACCAGGACTGGCCTAACTGTTGTAAGACAGT |
| pRK-ΔTPR7 | | Forward GATAAATCAGAAGCAAGTGCTGCAGCCTGGATGGACC  Reverse GGTCCATCCAGGCTGCAGCACTTGCTTCTGATTTATC |
| pRK-ΔTPR8 | | Forward ACAATTGGACCATGGCCATAATACCTCTGCACTTGC  Reverse GCAAGTGCAGAGGTATTATGGCCATGGTCCAATTGT |
| pET30c-G3BP1-NTF2L | | Forward AATCATATGGTCGGGCGGGAATTTGTG  Reverse AATGCGGCCGCGTATCTGAAGATATCATTGTG |
| pET30c-UTX-TPR | | Forward AATCATATGGAGTCTGATTTCTTTTGTCAA  Reverse AATGCGGCCGCCTAGTTGCATTTAAGTAGCA |

**Supplemental Table 2**. **qPCR primers used in this study**

| **Gene** | **Forward** | **Reverse** | |
| --- | --- | --- | --- |
| *G3BP1*  (H)  *UTX*  (H) | TGAGGTCTTTGGTGGGTTTG  GCTGGAACAGCTGGAAAGTC | | TGCTGTCTTTCTTCAGGTTCC  GAGTCAACTGTTGGCCCATT |
| *Actb* (H/M) | CCTCCCTGGAGAAGAGCTA | ACGTCACACTTCATGATGGA | |

**Supplemental Table 3**. **Antibodies used in this study**

| **Antibody** | **Company** | **Catalog number** |
| --- | --- | --- |
| UTX | Cell Signaling Technology | #33510 |
| UTX | Abcam | #ab36938 |
| G3BP1 | Abcam | #ab56574 |
| G3BP1 | Proteintech | #13057-2-AP |
| G3BP1 | Novus Biologicals | #NBP1-18922 |
| G3BP2 | Proteintech | #16276-1-AP |
| PABP | Abcam | #ab21060 |
| eIF3η  TIA1 | Santa Cruz  Santa Cruz | #sc-137215  #sc-166247 |
| USP10  CAPRIN1 | Proteintech  Proteintech | #19374-1-AP  #15112-1-AP |
| PDI  Ero1-L  H3K27me2  H3K27me3  Ac-H3K27  H3K4me3  5mC  GFP  Flag  Tubulin  β-actin | Cell Signaling Technology  Cell Signaling Technology  Abcam  PTM-Bio  Abcam  Cell Signaling Technology  Abcam  Proteintech  Sigma  Beyotime  Sigma | #2446  #3264  #24684  #622  #4729  #9751  #10805  #50430-2-AP  #F1804  #AF5012  #A5316 |
| H3 | ABclonal | #A2348 |

**Supplemental Table 4. Plasmids** used in this study

| **Plasmid** | **Source** |
| --- | --- |
| G3BP1-WT | This paper |
| G3BP1-NTF2L | This paper |
| G3BP1-Δ(RRM+RGG) | This paper |
| G3BP1-ΔNTF2L | This paper |
| G3BP1-RRM+RGG | This paper |
| Flag-ΔTPR2  Flag-ΔTPR5 | This paper  This paper |
| Flag-ΔTPR7 | This paper |
| Flag-ΔTPR8 | This paper |
| pET30c-TPR-6 × his | This paper |
| pET30c-NTF2L-6 × his | This paper |
| G3BP1-KO | This paper |
| G3BP2-KO | This paper |
| UTX-ΔTPR | MiaoLingBio (Wuhan, China) |
| UTX-ΔIDR  UTX-ΔJmjC  UTX-TPR | MiaoLingBio (Wuhan, China)  MiaoLingBio (Wuhan, China)  MiaoLingBio (Wuhan, China) |
| pEGFP-G3BP1 | MiaoLingBio (Wuhan, China) |
| UTX-D336G | Tsingke Biotechnology (Wuhan, China) |
| pFLAG-UTX  shUTX | Dr. Min Gyu Lee (MD Anderson Cancer Center)  Dr. Min Gyu Lee (MD Anderson Cancer Center) |
| UTX catalytic mutation | Dr. Min Gyu Lee (MD Anderson Cancer Center) |
| pddGFPB-C1  pddGFPA-G3BP1 | Liu lab (College of Life Sciences, Wuhan University)  Liu lab (College of Life Sciences, Wuhan University) |
| RFP-TIA1 | Liu lab (College of Life Sciences, Wuhan University) |


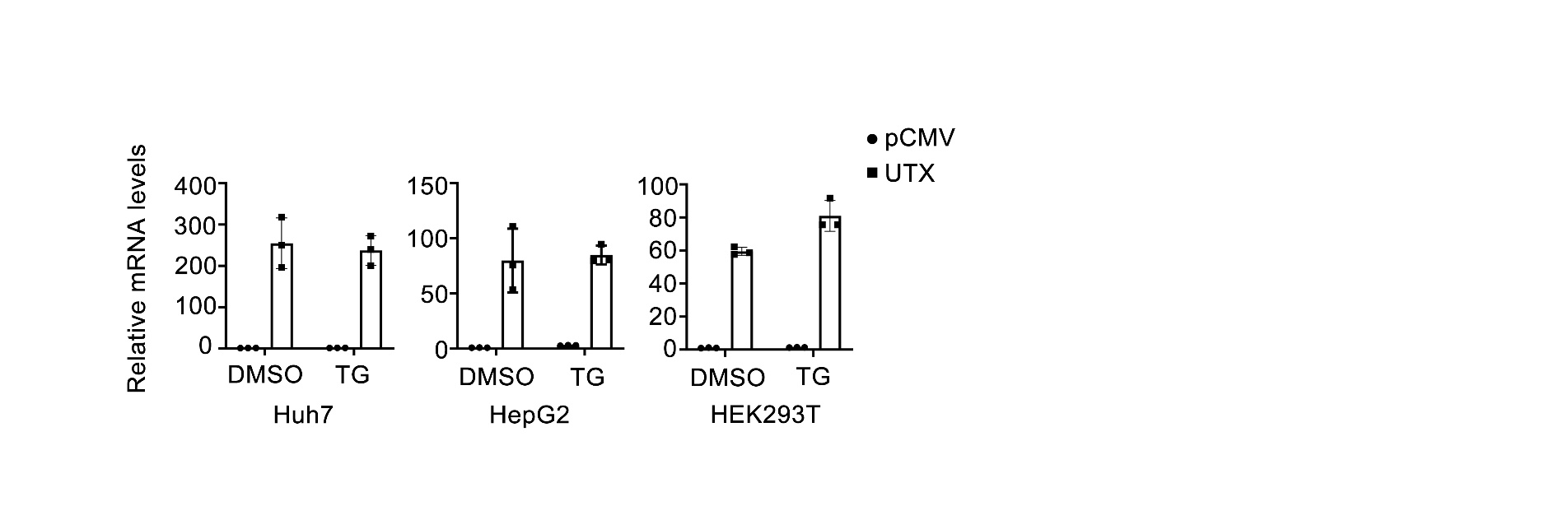


**Figure S1.** **Overexpression of UTX in multiple cell lines.**

qPCR results of *UTX* in Huh7 (left), HepG2 (middle) or HEK293T (right) cells transfected with empty vector (pCMV) or UTX^WT^ plasmid, with or without thapsigargin (TG) treatment (Huh7 cells, 1 μM TG, 1 hour; HepG2 cells, 5 μM TG, 4 hours; HEK293T cells, 2.5 μM TG, 1 hour). All results are representative for at least three independent experiments, with similar results obtained. Data are shown as the mean ± SD.


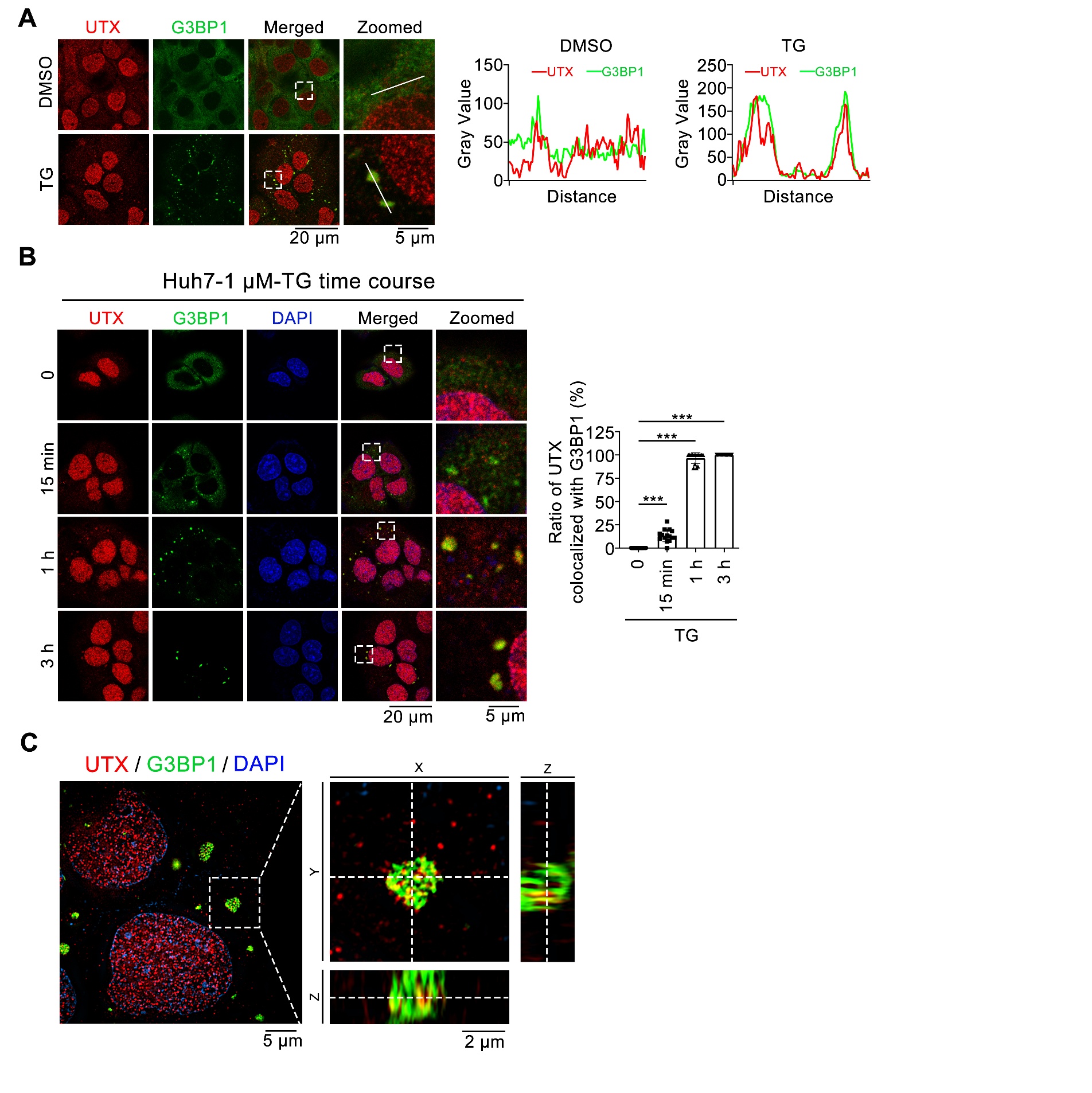


**Figure S2. A different commercial UTX antibody confirms that cytoplasmic UTX forms puncta and localizes in the stress granules of thapsigargin-treated cells.**

**A**) Representative images of endogenous UTX (red; antibody from CST) and G3BP1 (green; left panel) with plot analysis for the green and red pixel intensities along the indicated arrows in the zoomed pictures (right panel) in Huh7 cells treated with or without 1 μM TG for 1 hour. Scale bar, 20 μm. Magnification of the demarcated region was shown as the inset from the merged images, with the inset scale bar representing 5 μm. **B**) Representative images of endogenous UTX (red; antibody from CST) and G3BP1 (green; left panel) with quantification result of the percentage of cytoplasmic UTX puncta co-localized with G3BP1^+^ stress granules (right panel) in Huh7 cells treated with 1 μM TG for indicated times. Scale bar, 20 μm. Magnification of the demarcated region was shown as the inset for each condition from the merged images, with the inset scale bar representing 5 μm. Data are shown as the mean ± SD; n = 15 image fields per group from three different samples; ****P* < 0.001 (analyzed by one-way ANOVA). **C**) Z-stack projection of the representative image of co-localized endogenous UTX (red; an antibody from CST) and G3BP1 (green) in Huh7 cells under 1 μM TG treatment for 1 hour (left panel; scale bar, 5 μm); magnified orthogonal sectioning view of regions in the insert box (right panel; scale bar, 2 μm). DAPI, blue, stained nuclei. All results are representative for at least three independent experiments, with similar results obtained.


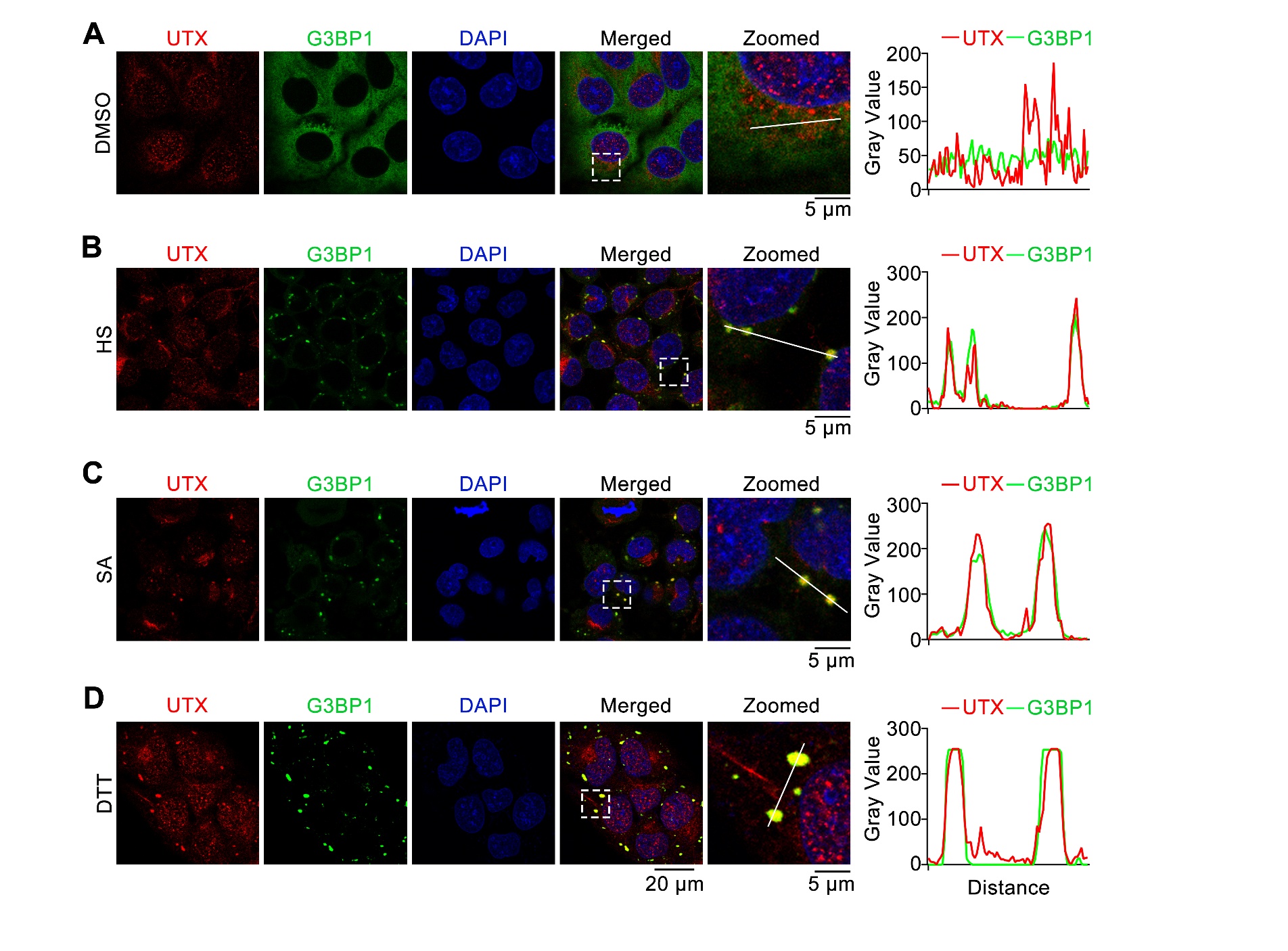


**Figure S3. Cytoplasmic UTX puncta co-localize with SGs upon heat shock-, sodium arsenite-, or DTT-induced stresses.**

**A-D**) Representative images of endogenous UTX (red) and G3BP1 (green) in Huh7 cells treated with vehicle (DMSO; **A**), heat shock (HS; **B**), 500 μM sodium arsenite (SA; **C**), or 1 mM DTT (**D**) for 1 hour (left panel) with plot analysis of the red and green pixel intensities along the indicated arrows in the zoomed pictures (right panel). Scale bar, 20 μm. Magnification of the demarcated region was shown as the inset for each condition from the merged images, with inset scale bars representing 5 μm. All results are representative for at least three independent experiments, with similar results obtained.


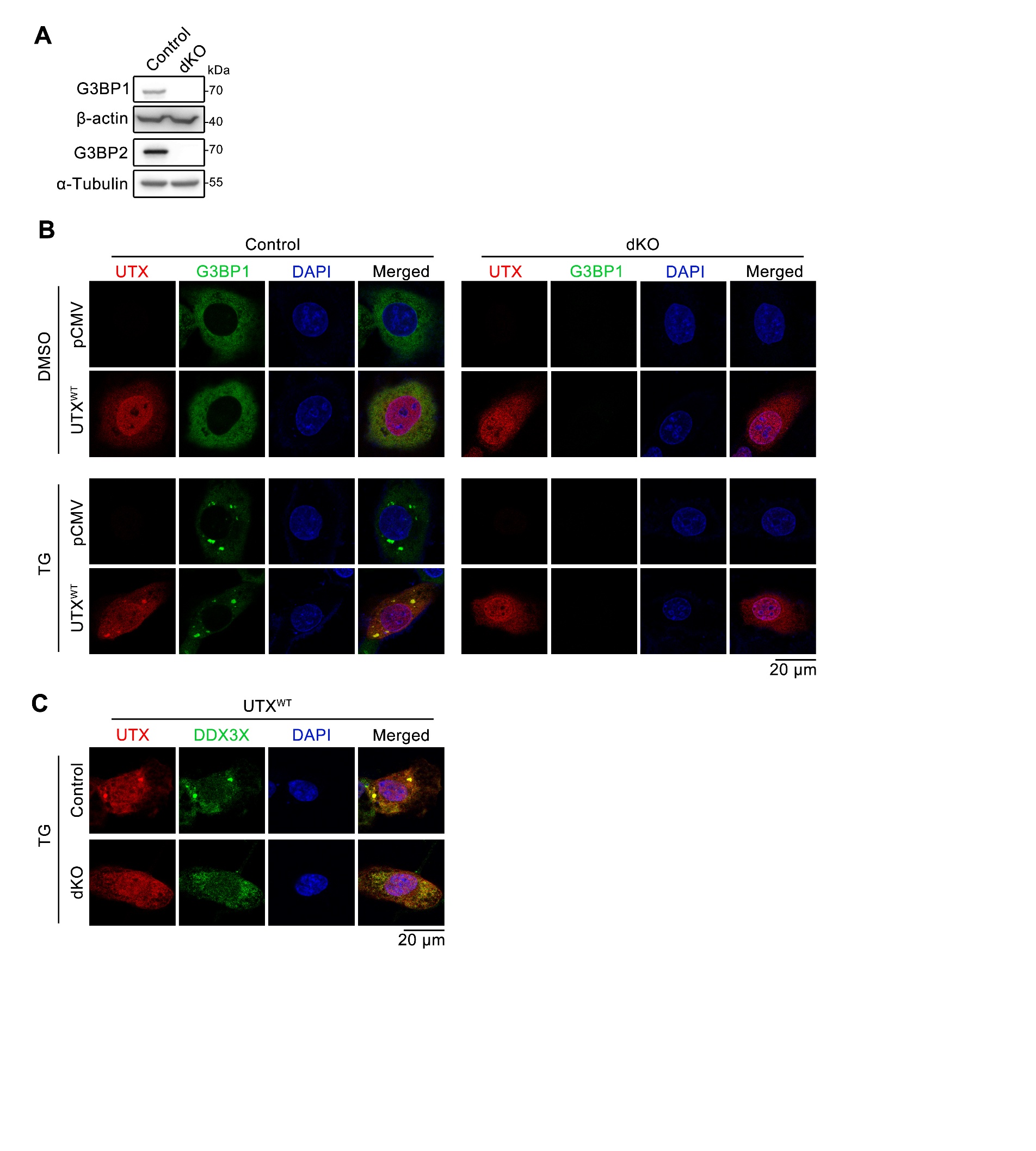


**Figure S4. Formation of cytoplasmic UTX puncta depends on stress granules.**

**A**) Knockout efficiency for stable dKO U2OS cells. **B**) Representative images of UTX (red) and G3BP1 (green) in stable dKO U2OS cells transfected with empty vector (pCMV) or UTX WT plasmid (UTX^WT^) treated with vehicle (DMSO) or 5 μM TG for 1 h. Scale bar, 20 μm. **C**) Representative images of UTX (red) and another SG marker DDX3X (green) in stable dKO U2OS cells transfected with UTX^WT^, and treated with 5 μM TG for 1 h. Scale bar, 20 μm. Results are representative for at least three independent experiments, with similar results obtained.


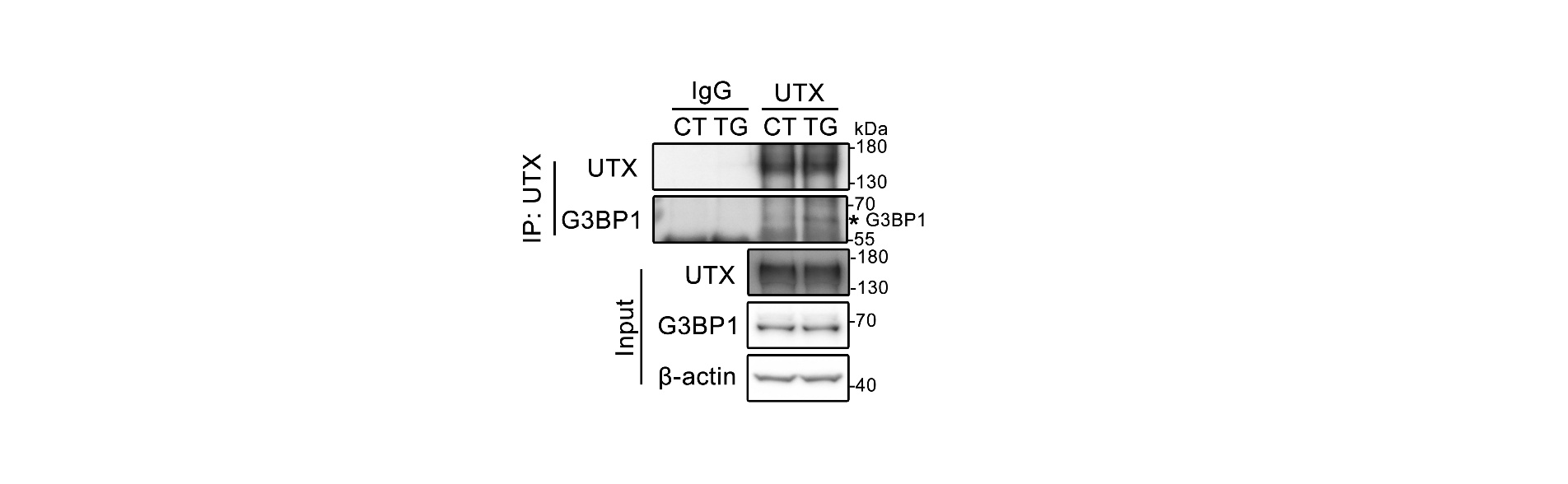


**Figure S5. UTX interacts with G3BP1 in primary mouse hepatocytes.**

Co-immunoprecipitation assays for UTX with G3BP1 in primary mouse hepatocytes with DMSO (CT) or 10 μM TG treatment for 1 hour. * indicates corresponding band of G3BP1. Results are representative for at least three independent experiments, with similar results obtained.


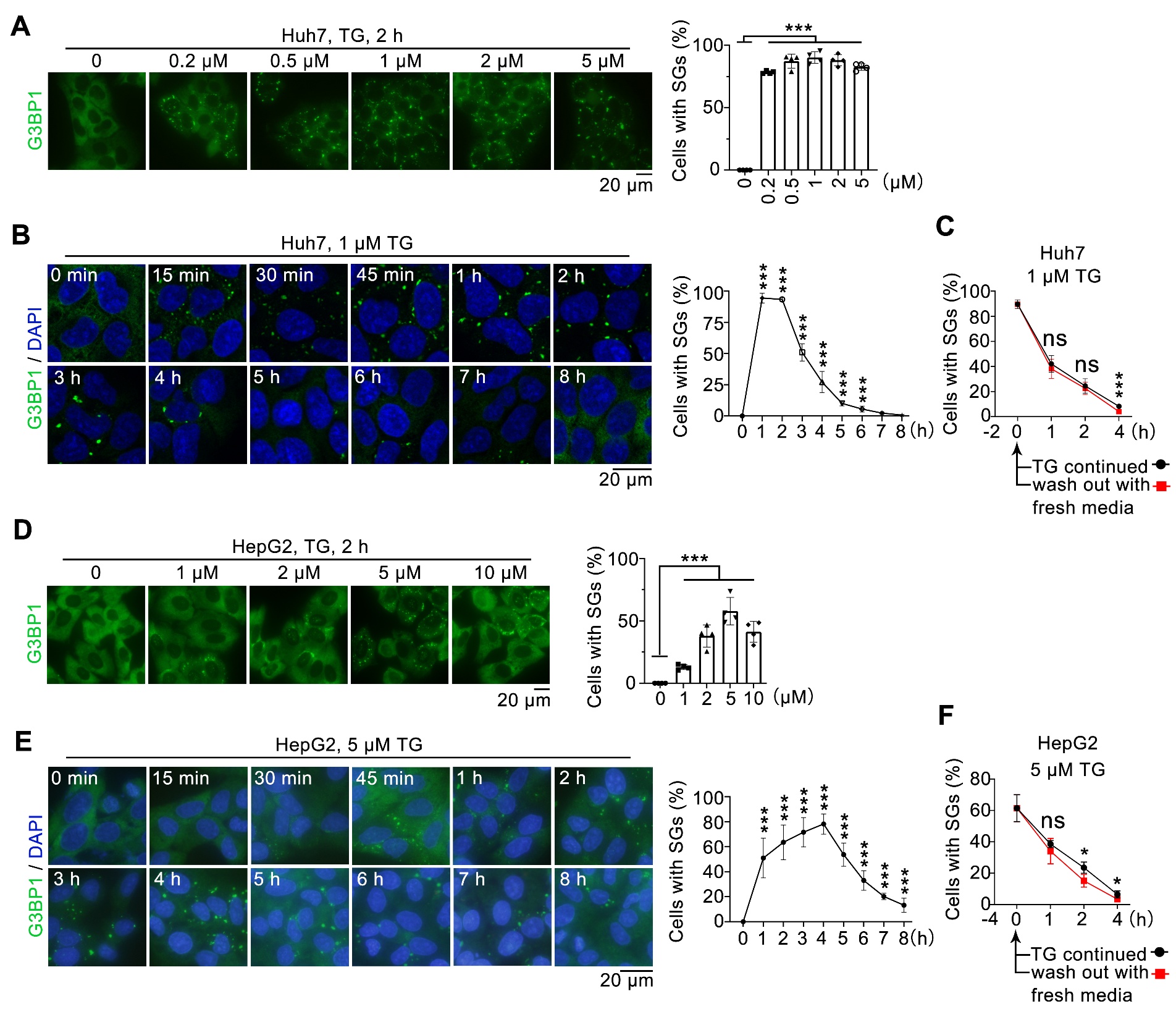


**Figure S6. Time dependent assembly and disassembly of stress granules upon thapsigargin treatment.**

**A-B**) Representative images (left) with quantitative results (right) for the percentage of cells with G3BP1 puncta (green) in Huh7 cells treated with indicated concentrations of TG for 2 hours (**A**) or 1 μM TG for indicated time (**B**). Scale bar, 20 μm. n = 4 image fields per group. **C**) The percentage of cell with SGs in Huh7 cells pretreated with 1 μM TG for 2 hours, then with continuous TG treatment or TG washed out with fresh media for indicated time. n = 6 image fields per group. **D-E**) Representative images (left) with quantitative results (right) for the percentage of cells with G3BP1 puncta (green) in HepG2 cells treated with indicated concentrations of TG for 2 hours (**D**) or 5 μM TG for indicated time (**E**). Scale bar, 20 μm. n = 4 image fields per group. **F**) The percentage of cell with SGs in HepG2 cells pretreated with 5 μM TG for 4 hours, then with continuous TG treatment or TG washed out with fresh media for indicated time. n = 4 image fields per group. All results are representative for at least three independent experiments, with similar results obtained. Data are shown as the mean ± SD, and analyzed by one-way ANOVA (for **A**, **D**) or analyzed by two-tailed Student’s t-test for (for **B**-**C** and **E**-**F**). **P* < 0.05, ***P* < 0.01, ****P* < 0.001; ns, not significant.


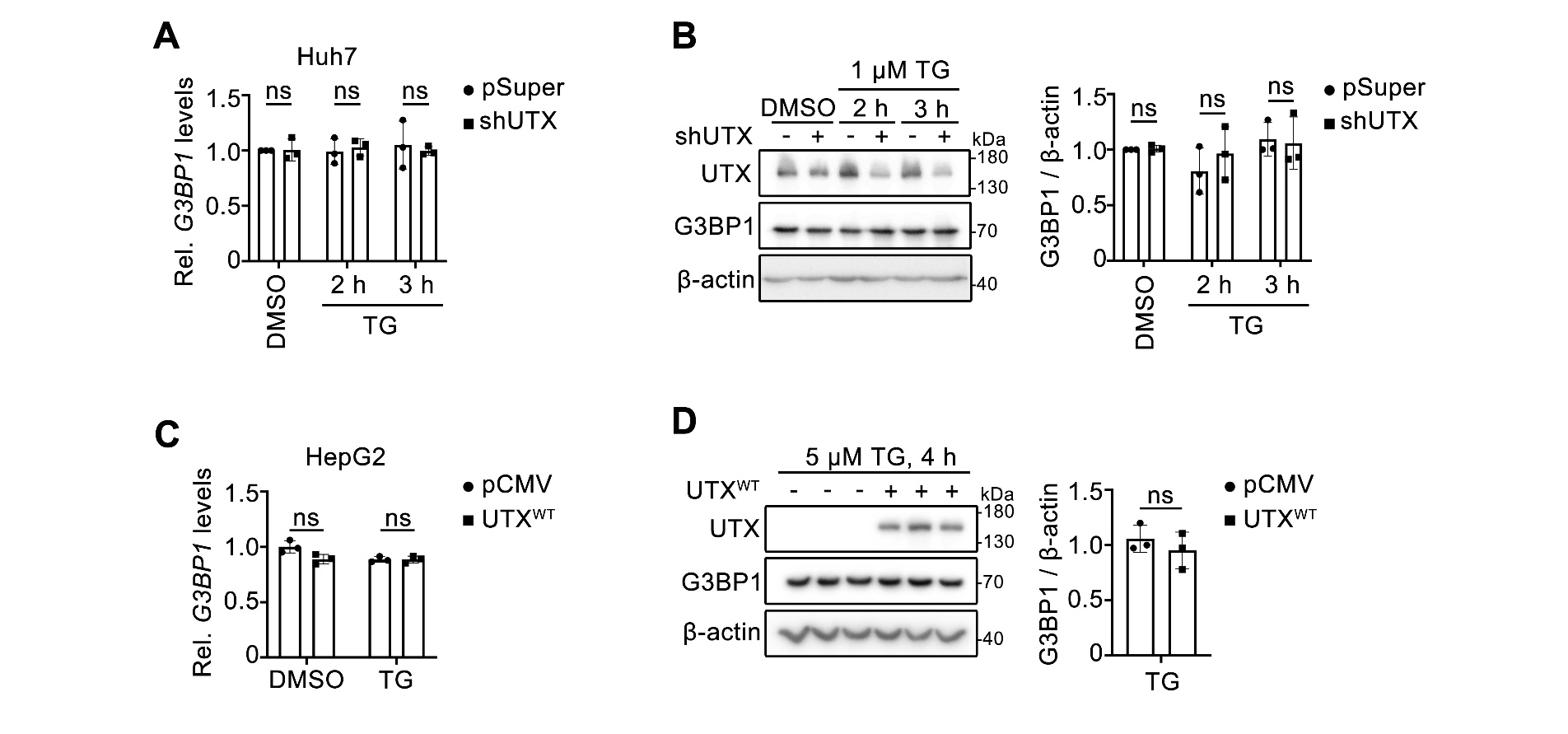


**Figure S7. UTX does not affect the transcription and protein levels of G3BP1.**

**A-B**) qPCR results (**A**) and representative Western blots (**B**, left) with quantitative results (**B**, right) of G3BP1 in Huh7 cells transfected with the control (pSuper) or shUTX under 1 μM TG treatment for indicated time. **C-D**) qPCR results (**C**) and representative Western blots (**D**, left) with quantitative results (**D**, right) of G3BP1 in HepG2 cells transfected with empty vector (pCMV) or UTX^WT^ plasmid at 4 hours after 5 μM TG treatment. All results are representative for at least three independent experiments, with similar results obtained. Data are shown as the mean ± SD, and analyzed by two-tailed Student’s t-test. ns, not significant.


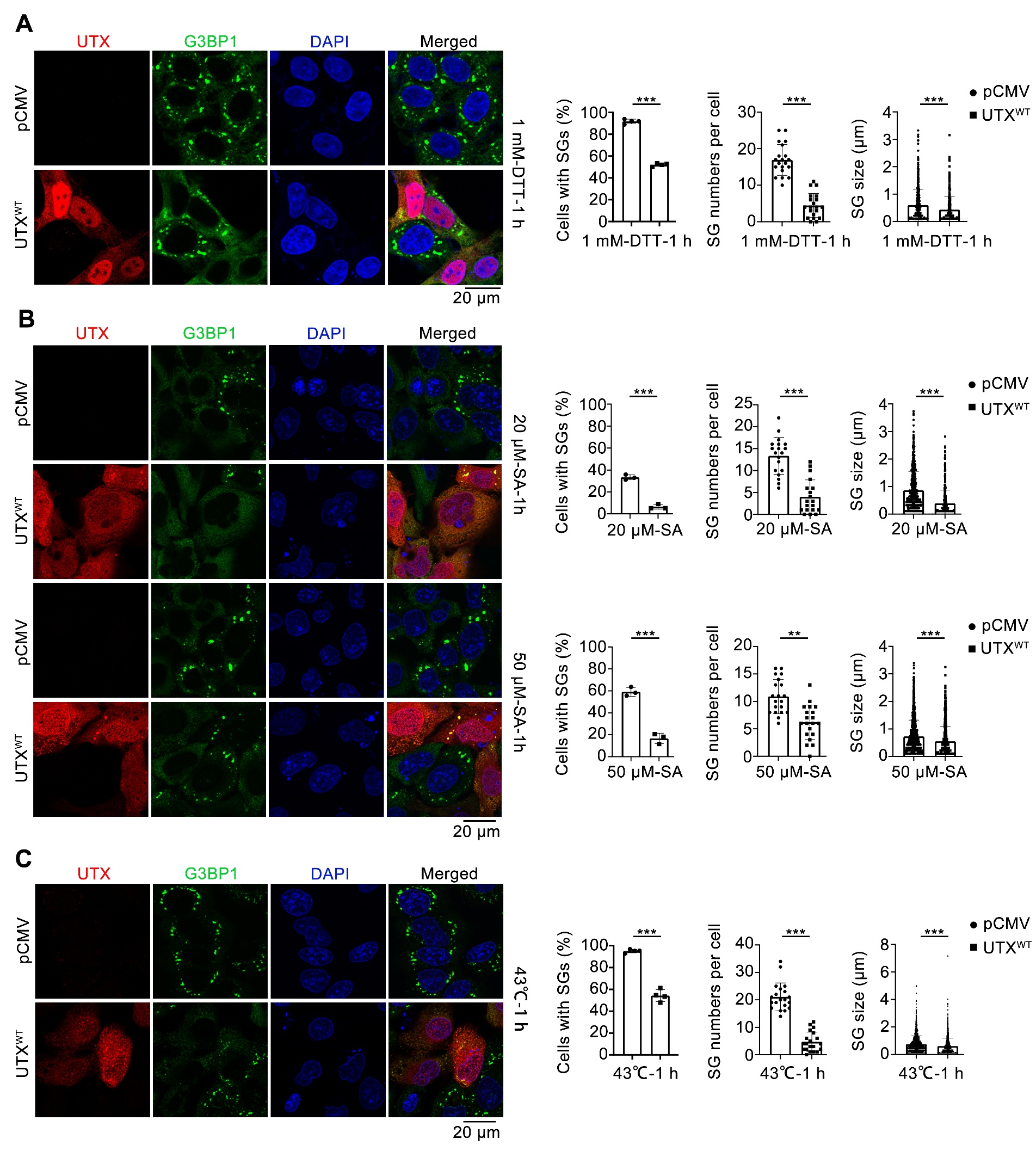


**Figure S8. The effects of UTX on stress granule are also found in DTT, sodium arsenite, and heat shock treatments.**

**A**) Representative images of UTX (red) and G3BP1 (green) in HepG2 cells transfected with empty vector (pCMV) or UTX wildtype (UTX^WT^) plasmid under 1 mM DTT treatment for 1 hour. Scale bar, 20 μm. Quantitative results of the percentage of cells with SG (left, n = 4 independent samples per group), number of SG per cell (middle, n = 20 cells per group collected from four independent samples), and SG size (right, 12 images per group collected from four independent samples). **B**) Representative images of UTX (red) and G3BP1 (green) in HepG2 cells transfected with empty vector (pCMV) or UTX wildtype (UTX^WT^) plasmid under 20 μM or 50 μM SA treatment for 1 hour. Scale bar, 20 μm. Quantitative results of the percentage of cells with SG (left, n = 3 independent samples per group), number of SG per cell (middle, 20 μM, n = 19 cells; 50 μM, n = 20 cells collected from three independent samples), and SG size (right, 12 images per group collected from three independent samples). **C**) Representative images of UTX (red) and G3BP1 (green) in HepG2 cells transfected with empty vector (pCMV) or UTX wildtype (UTX^WT^) plasmid under 43 ℃ treatment for 1 hour. Scale bar, 20 μm. Quantitative results of the percentage of cells with SG (left, n = 4 independent samples per group), number of SG per cell (middle, n = 20 cells per group collected from four independent samples), and SG size (right, 12 images per group collected from four independent samples). All results are representative for at least three independent experiments, with similar results obtained. Data are shown as the mean ± SD, and analyzed by two-tailed Student’s t-test. ***P* < 0.01, ****P* < 0.001.


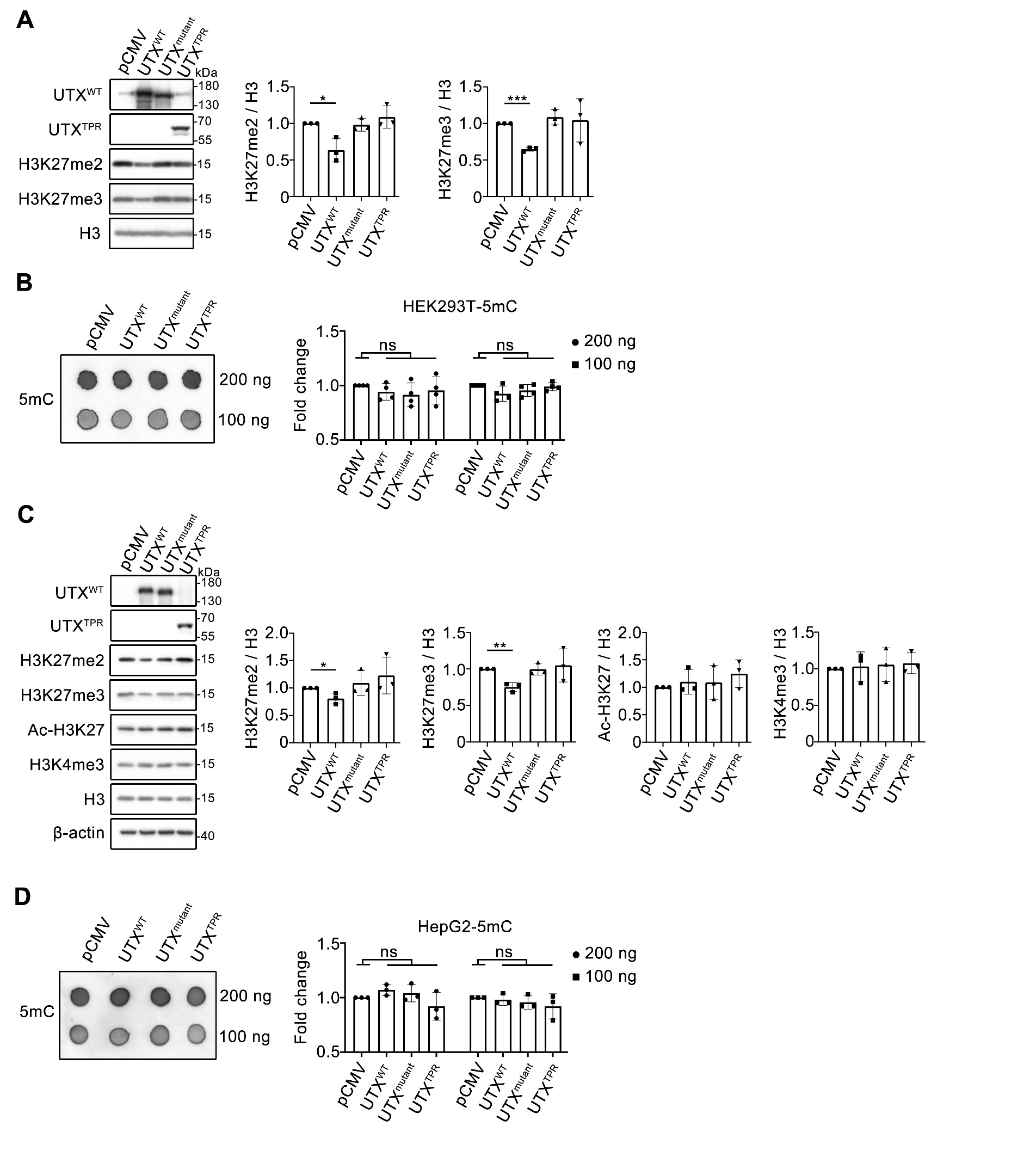


**Figure S9. The effects of UTX^WT^, UTX^mutant^, or UTX^TPR^ on total histone methylation and DNA methylation levels.**

**A**) Representative western blots (left) with quantitative results (right) of H3K27me2 and H3K27me3 in HEK293T cells transfected with empty vector (pCMV), UTX^WT^, UTX^mutant^, or UTX^TPR^ plasmids. **B**) Dot blot assay of 5mC levels in HEK293T cells transfected with empty vector (pCMV), UTX^WT^, UTX^mutant^, or UTX^TPR^ plasmids. **C**) Representative Western blots (left) with quantitative results (right) of H3K27me2, H3K27me3, H3K37ac and H3K4me3 in HepG2 cells transfected with empty vector (pCMV), UTX^WT^, UTX^mutant^, or UTX^TPR^ plasmids. **D**) Dot blot assay of 5mC levels in in HepG2 cells transfected with empty vector (pCMV), UTX^WT^, UTX^mutant^, or UTX^TPR^ plasmids. All results are representative for at least three independent experiments, with similar results obtained. Data are shown as the mean ± SD, and analyzed by one-way ANOVA. **P* < 0.05, ***P* < 0.01, ****P* < 0.001; ns, not significant.


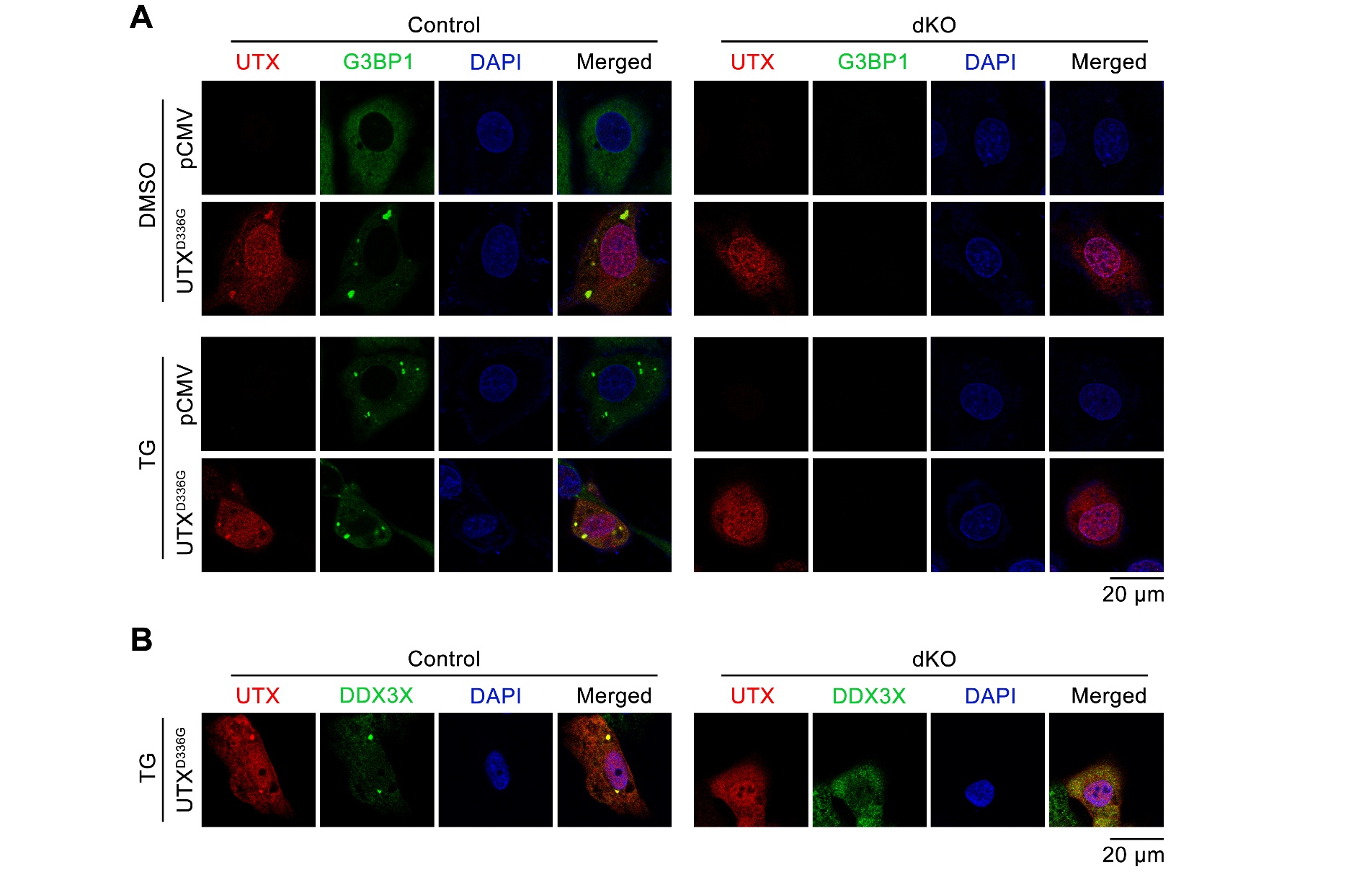


**Figure S10. Stable formation of cytoplasmic puncta for UTX^D336G^ depends on the existence of stress granules.**

**A**) Representative images of UTX (red) and G3BP1 (green) in stable dKO U2OS cells transfected with empty vector (pCMV) and UTX^D336G^, respectively, treated with vehicle (DMSO) or 5 μM TG for 1 hour. Scale bar, 20 μm. **B**) Representative images of UTX (red) and another SG marker DDX3X (green) in stable dKO U2OS cells that transfected with UTX^D336G^ and treated with 5 μM TG for 1 hour. Scale bar, 20 μm. Results are representative for at least three independent experiments, with similar results obtained.


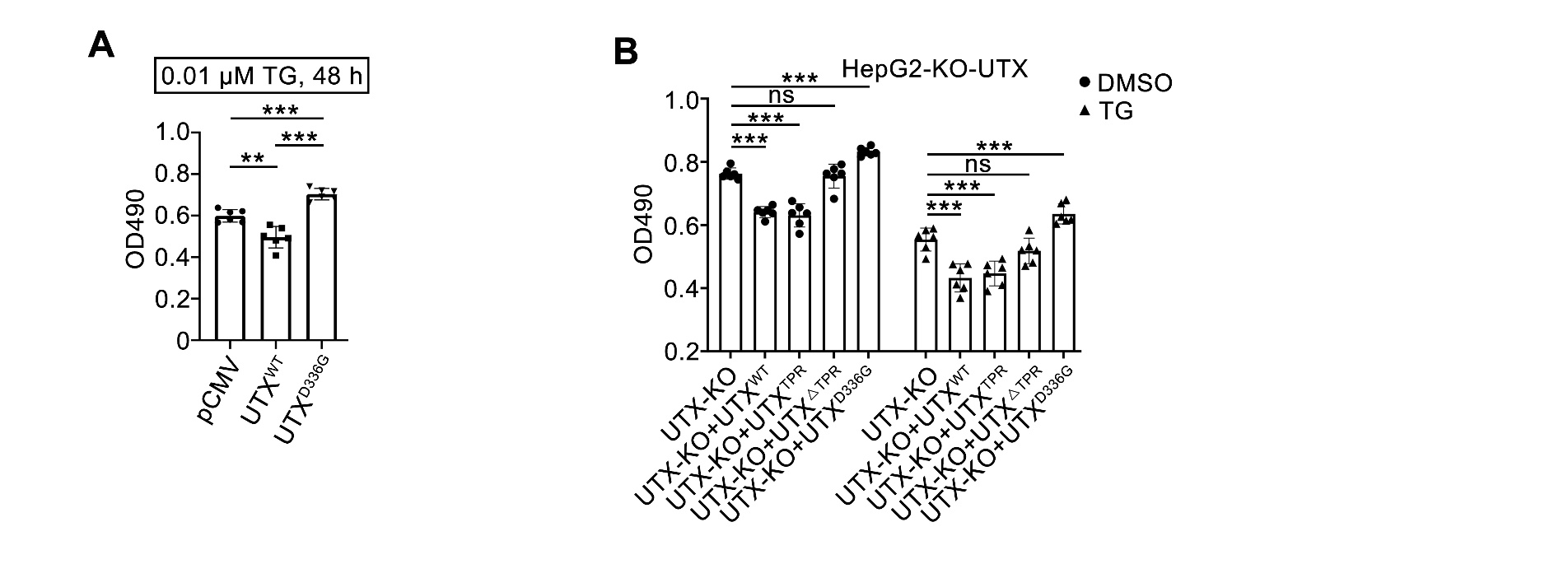


**Figure S11. MTT assays in WT or stable UTX knockout HepG2 cells.**

**A**) MTT assays in HepG2 cells transfected with empty vector (pCMV), UTX^WT^, or UTX^D336G^ under 0.01 μM TG treatment for 48 hours. n = 6 per group. **B**) MTT assays in stable UTX knockout HepG2 cells transfected with empty vector (pCMV), UTX^WT^, UTX^TPR^, UTX^△TPR^, or UTX^D336G^ under vehicle (DMSO) or 5 μM TG treatment for 4 hours. n = 6 per group. All results are representative for at least three independent experiments, with similar results obtained. Data are shown as the mean ± SD, and analyzed by one-way ANOVA. ***P* < 0.01, ****P* < 0.001; ns, not significant.


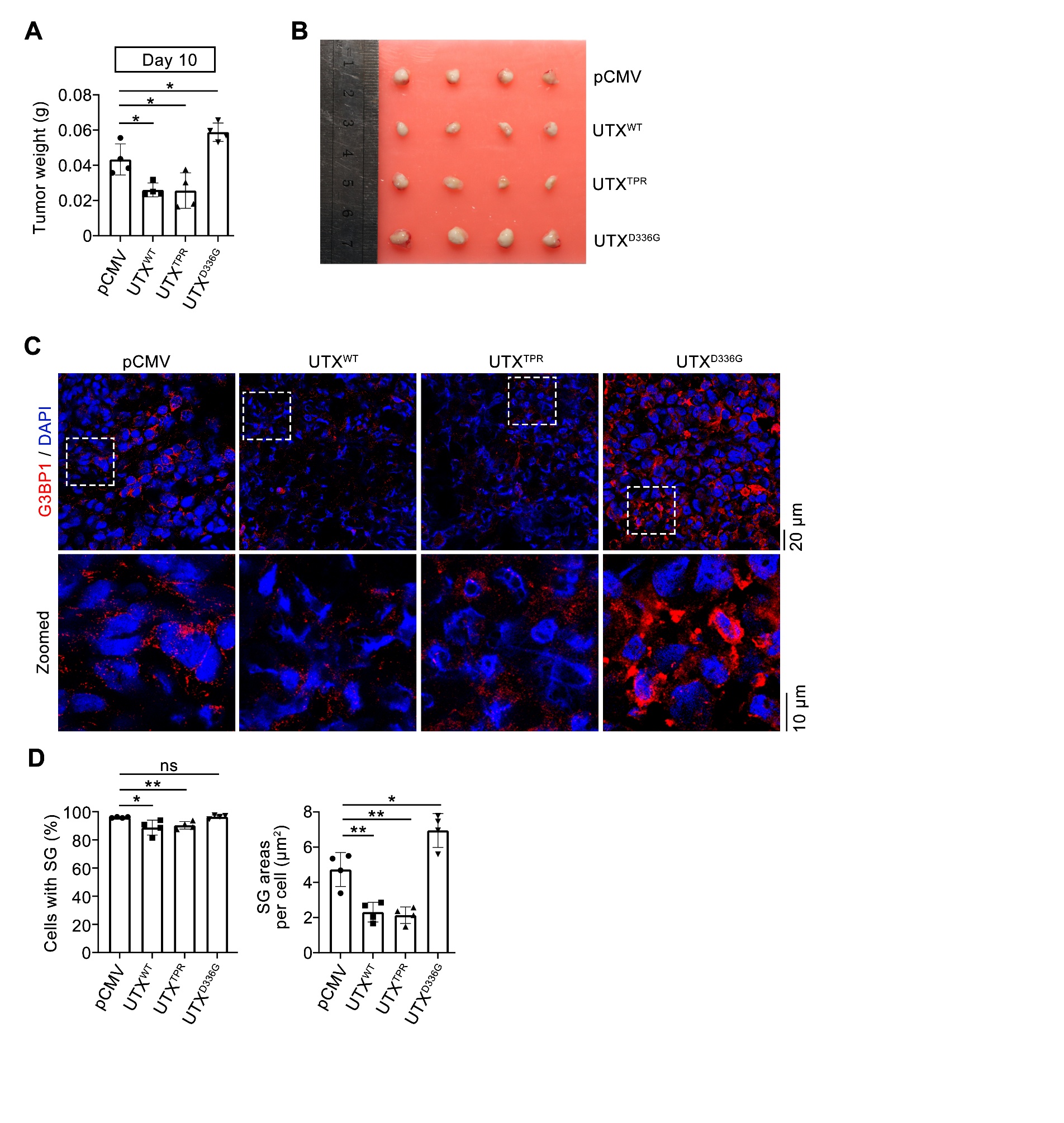


**Figure S12. The effects of UTX^WT^, UTX^TPR^ and UTX^D336G^ on xenograft tumor growth at day 10 after cell injection.**

**A-B**) Weights of xenograft tumors (**A**) with images for each xenograft tumor isolated from nude mice (**B**) at Day10 for indicated groups. pCMV, n = 4; UTX^WT^, n = 4; UTX^TPR^, n = 4; UTX^D336G^, n = 4. **C-D**) Representative images of G3BP1 staining (**C**) with quantitative results of the percentage of cells with SGs (**D**, left) and SG areas per cell (**D**, right) in xenograft tumors. G3BP1^+^, red; DAPI stained nuclei, blue. n = 4 mice per group. Scale bar, 20 μm. Shown also are magnifications of indicated inset regions, with the inset scale bar representing 10 μm. Data are shown as the mean ± SD, and analyzed by one-way ANOVA. **P* < 0.05, ***P* < 0.01; ns, not significant.


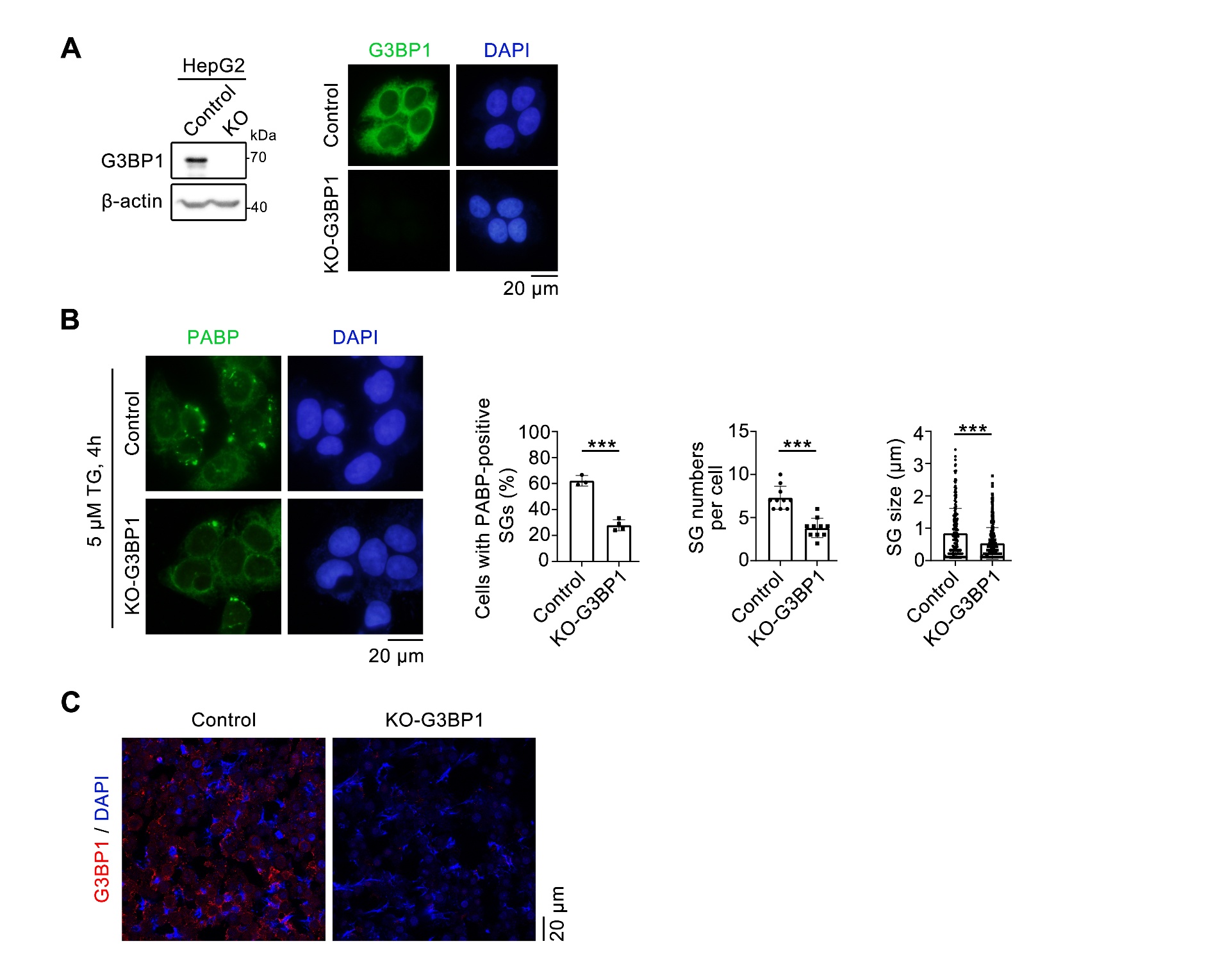


**Figure S13. Verification of G3BP1 knockout in HepG2 cells and nude mice.**

**A**) Knockout efficacy of stable G3BP1 knockout HepG2 cells with Western blots (left) and immunofluorescent staining (right). G3BP1^+^, green; DAPI stained nuclei, blue. **B**) Representative images of PABP (green) (left) with quantitative results (right) in stable G3BP1 knockout HepG2 cells treated with 5 μM TG for 4 hours. Scale bar, 20 μm. The percentage of cells with SG (control, n = 3; KO-G3BP1, n = 4), number of SG per cell (n = 10 cells per group collected from three independent samples), and SG size (9 images per group collected from three independent samples). **C**) Representative images of G3BP1 staining in xenograft tumors after injecting G3BP1 knockout HepG2 cells. G3BP1^+^, red; DAPI stained nuclei, blue. Scale bar, 20 μm. All results are representative for at least three independent experiments, with similar results obtained. Data are shown as the mean ± SD, and analyzed by two-tailed Student’s t-test. ****P* < 0.001.
